# An Activity‐Dependent NEPAS–PTX3 Axis Links Neurovascular and Myelin Deficits to Cognitive Impairment

**DOI:** 10.1002/advs.202521069

**Published:** 2026-04-07

**Authors:** Boya Hu, Zifei Chen, Bingmei Sun, Jiale Gao, Jiale Xu, Xiaochun Guo, Fenfei Gao, Zhongsi Wang, Jie Wu, Xiaoyu Ji, Peipei Liu, Bing Huang

**Affiliations:** ^1^ Department of Neurosurgery The First Affiliated Hospital of Shantou University Medical College Shantou Guangdong China; ^2^ Pain and Related Diseases Research Laboratory Shantou University Medical College Shantou Guangdong China; ^3^ Department of Pharmacology Shantou University Medical College Shantou Guangdong China; ^4^ Beijing SeqWisdom Biotechnology Co., Ltd. Beijing China; ^5^ Clinical Systems Biology Laboratories The First Affiliated Hospital of Zhengzhou University Zhengzhou Henan China

**Keywords:** cognitive deficits, medial prefrontal cortex (mPFC), myelination, NEPAS, neurovascular coupling, pentraxin‐3 (PTX3)

## Abstract

Acquired cognitive impairments occur in diverse neurological and psychiatric conditions, yet the mechanisms linking neural circuit activity to neurovascular function remain poorly understood. Using a mouse model of nicotine withdrawal (WD), we identified an activity‐dependent NEPAS–pentraxin‐3 (PTX3) axis in mPFC neurons that couples circuit hypoactivity to cognitive deficits. In this model, NEPAS expression is significantly upregulated in mPFC neurons, accompanied by reduced myelin formation. Neuronal activity in mPFC and PVA is suppressed and chemogenetic activation of the PVA–mPFC neural circuit downregulates NEPAS. Elevated neuronal NEPAS suppresses the secretion of PTX3, thereby impairing angiogenesis. Conversely, knockdown of neuronal NEPAS restores PTX3 expression and angiogenesis, alleviates myelin formation deficits, and improves cognitive memory following nicotine WD. Notably, activation of the PVA–mPFC neural circuit produces similar therapeutic effects. Human transcriptomic data reveal a consistently elevated HIF‐3α expression in ex‐smokers versus controls. Our findings demonstrate that the NEPAS–PTX3 axis in mPFC neurons links neural circuit hypoactivity to neurovascular and myelin deficits, providing a mechanistic framework for acquired cognitive impairment. This pathway represents a potential target for neuromodulation‐based therapies in prefrontal circuit dysfunction associated cognitive disorders.

## Graphical abstract

An activity‐dependent pathway links prefrontal circuit hypoactivity to cognitive impairment. Reduced PVA–mPFC activity upregulates NEPAS, which suppresses PTX3 secretion, leading to impaired angiogenesis, myelin deficits, and memory decline. Rescue is achieved by NEPAS knockdown or chemogenetic circuit activation. This NEPAS–PTX3 axis reveals a reversible mechanism connecting neural activity to brain homeostasis, offering a target for cognitive disorders.

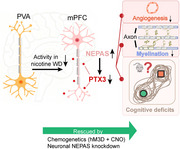

## 1 Introduction

Acquired cognitive impairments are a major source of functional disability in diverse neurological and psychiatric disorders, including traumatic brain injury, post‐chemotherapy states, and substance use disorders [1]. Tobacco use is a leading contributor to substance use disorders. Notably, smoking cessation correlates with cognitive decline in abstinent individuals, particularly working memory deficits [2, 3, 4, 5]. This phenomenon has been consistently recapitulated in animal models [6]. A common neurobiological basis for these impairments lies in dysfunction of the medial prefrontal cortex (mPFC). mPFC is critical for executive function, working memory, and behavioral flexibility. While existing research has thoroughly characterized the complex effects of acquired cognitive impairments, the underlying neurobiological mechanisms remain poorly understood.

Cognitive function relies on the coordinated activity of neurons, glia, and the cerebrovascular system. Adequate cerebral blood flow and oxygen delivery are essential for sustaining neuronal metabolism, synaptic plasticity, and myelination. These processes are mediated by angiogenesis and neurovascular coupling [7]. Disruption of angiogenesis and vascular rarefaction are increasingly recognized as contributors to cognitive disorders, including aging related dementia, Alzheimer's disease (AD), and vascular cognitive impairment (VCI) [8, 9, 10]. Neurons actively regulate cerebral angiogenesis through releasing angiogenesis factors or neurotransmitters. This regulation involves multiple signaling pathways. For example, neuronal release of vascular endothelial growth factor (VEGF) promotes vascular formation by binding to tyrosine kinase receptors on endothelial cells [11, 12]. S. Biswas et al. demonstrated that glutamatergic neuronal activity modulates retinal angiogenesis via the Norrin/β‐catenin pathway [13]. Moreover, direct neuron‐vessel communication has been identified. It is achieved via synapse‐like structures formed by neuron‐astrocyte connections with vascular smooth muscle cells [14]. However, the molecular mechanisms by which neuronal activity regulates angiogenesis in the brain, the involvement of neural circuits in this process, and their regulatory roles in cognition remain poorly understood [11].

Hypoxia‐inducible factors (HIFs) are key transcription factors that respond to hypoxic stress. Among HIF isoforms, HIF‐1α and HIF‐2α are well characterized and structurally homologous. HIF‐3α is evolutionarily distant and understudied [15]. In contrast to the pro‐angiogenic role of HIF‐1α and HIF‐2α via VEGF, HIF‐3α exerts dual effects on angiogenesis. It can either promote or inhibit angiogenesis depending on its isoform [16]. HIF‐3α generates three distinct isoforms: classical HIF‐3α, NEPAS, and the shortest variant, IPAS [16]. Studies have indicated that NEPAS and IPAS suppress HIF‐1α‐ and HIF‐2α‐mediated gene expression by competing for DNA binding sites, thereby influencing the hypoxic response during vascular development [17]. Dysregulated HIF‐3α has been linked to pathological conditions such as malignant meningioma [18], impaired lung remodeling [19], and distal arthrogryposis type 2B (DA2B) [20]. Notably, although HIF‐3α is expressed in various cell types [21], most studies have focused on its endothelial‐intrinsic role. Its function in non‐endothelial cells, particularly in neurons, and its potential involvement in neurovascular coupling during behavioral states remain largely unexplored.

The mPFC is a critical hub for decision‐making and working memory. It maintains extensive connections to other brain regions [22]. A main glutamatergic input to the mPFC originates from the paraventricular thalamic nucleus (PVT), and neuronal activity within this circuit plays a crucial role in learning and memory [23, 24]. PVT is part of the midline and intralaminar thalamic nuclei and is well known for its role in regulating circadian rhythms and wakefulness [25]. Anatomically, the PVT is divided into anterior and posterior regions, referred to as the anterior paraventricular thalamic nucleus (aPVT or PVA) and posterior paraventricular thalamic nucleus (pPVT or PVT), respectively [26]. PVT composed of divergently projecting neurons which innervate the limbic striatal and cortical regions, one of which is the mPFC [27]. Studies have shown that the PVA plays a regulatory role in chronic muscle pain [28], chronic mechanical hyperalgesia [29], and neuropathic pain‐induced cardioprotection [30]. However, unlike the pPVT, which is involved in a wide range of physiological processes and has well‐defined functions, the role of the PVA remains poorly studied and understood. In particular, the functional impact of the PVA–mPFC neural circuit on cognitive memory and the underlying mechanisms remain unclear.

In this study, we combined a mouse model of cognitive impairment with transcriptomic profiling and cell‐type‐specific genetic manipulations to investigate the role of neuronal NEPAS in neurovascular dysfunction. We demonstrated that activity‐regulated neuronal NEPAS impairs myelination and cognitive memory by inhibiting neuronal release of PTX3 and subsequently suppressing angiogenesis. We further explored the influence of PVA–mPFC neural circuit activity on NEPAS‐mediated regulation of angiogenesis and cognitive memory. Our findings reveal a previously unknown activity‐dependent NEPAS–PTX3 axis in the mPFC, linking neural circuit hypoactivity to cognitive impairment through NEPAS‐mediated suppression of angiogenesis.

## 2 Results

### 2.1 Nicotine WD Upregulates Neuronal NEPAS and Impairs Myelination in the mPFC

**FIGURE 1:**
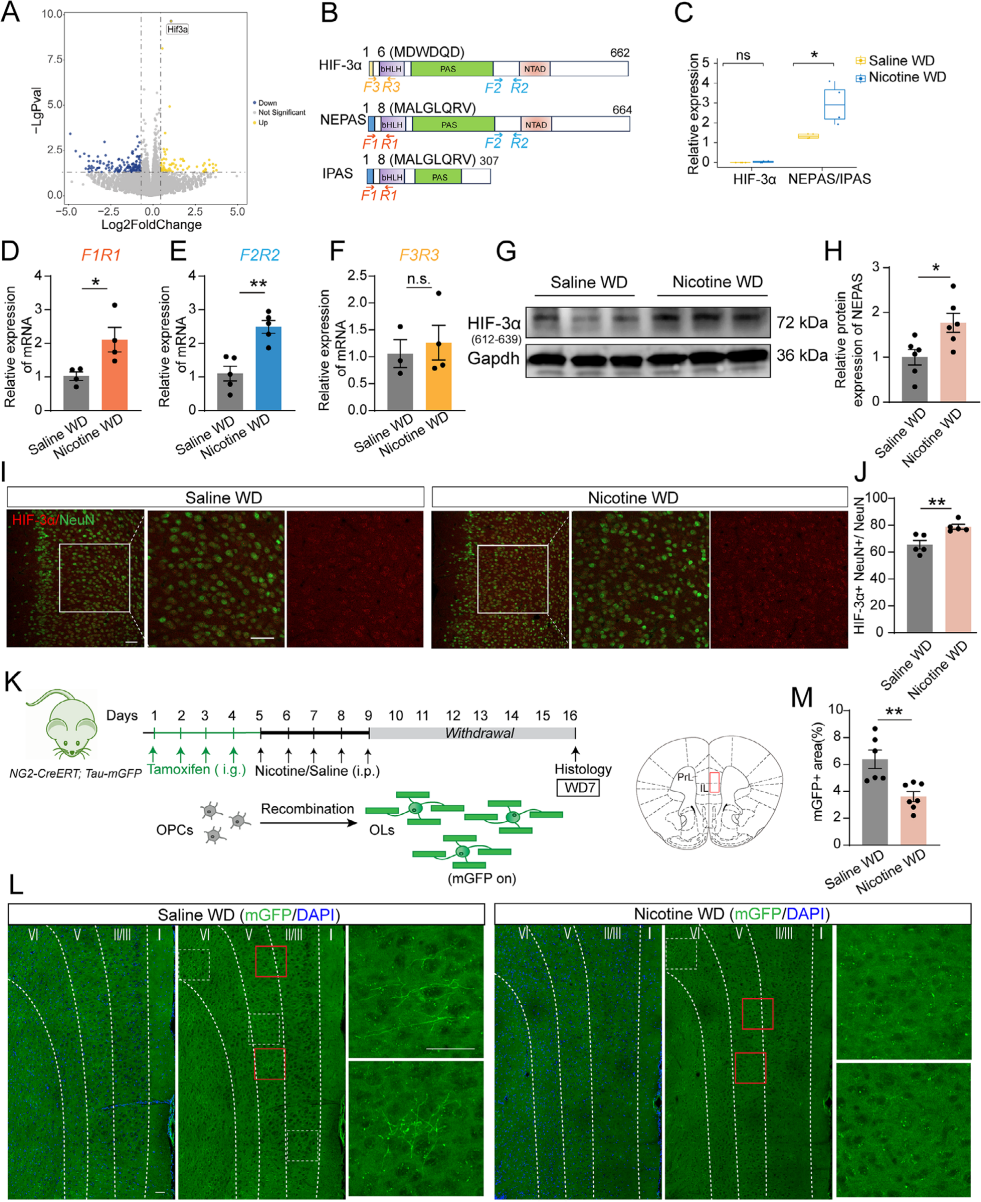
Nicotine WD caused increased NEPAS in neurons and decreased myelin formation in the mPFC. (A) Volcano plot showed DEGs identified by RNA sequencing between nicotine WD and saline WD, with *Hif3a* showing the most significant upregulation. (B) Structures of three distinct protein isoforms of *Hif3a* and primers for RT‐qPCR. Both classical HIF‐3α and NEPAS contain bHLH, PAS and NTAD domains, whereas IPAS lacks the NTAD domain. The amino acid sequence between NEPAS and HIF‐3α is similar, with HIF‐3α starting with MDWDQD and NEPAS with MALGLQRV. (C) Isoform analysis from RNA sequencing FPKM showed the NEPAS/IPAS was the most significant upregulated isoforms (HIF‐3α: n = 4 for each group, *P* = 0.134; NEPAS/IPAS: n = 4 for each group, ^*^ *P* < 0.05, two‐tailed Student's t test). (D–F) Transcription levels of distinct isoforms showed by RT‐qPCR. Sequences targeted by primers F1R1 and F2R2 were upregulated (D,E) and that targeted by F3R3 was not altered (F1R1: n = 4 for each group, ^*^ *P* < 0.05; F2R2: n = 5 for each group, ^**^ *P* < 0.01, two‐tailed Student's *t*‐test). (G) Protein expression level of HIF‐3α and NEPAS by blotted with a monoclonal antibody targeted to N‐terminal (612‐639) of HIF‐3α, which was lack in IPAS isoform. (H) Expression of NEPAS was significantly increased after nicotine WD (n = 6 for each group; ^*^ *P* < 0.05, two‐tailed Student's t test). (I,J) Immunofluorescence images staining showed an increase of HIF‐3α in the mPFC neurons after nicotine WD (n = 5 for each group; ^**^ *P* < 0.01, two‐tailed Student's t test). (K) Schematic diagram displaying the time course of tamoxifen induction and histological region after nicotine WD procedure in the NG2‐CreERT; Tau‐mGFP mice. Newly formed myelin sheath specifically during nicotine WD will be labeled with mGFP signals. (L) Representative images showing newly formed myelin during Saline WD or Nicotine WD in the mPFC. (M) Statistic results showed a significant decrease of mGFP positive areas in the Nicotine WD group (n = 6, 7 for Saline WD and Nicotine WD, respectively; ^**^ *P* < 0.01, two‐tailed Student's t test). Scale bars, 50 µm. Data are presented as mean ± SEM.

In a nicotine WD caused cognitive deficits model [6], we performed transcriptomic analysis of mPFC after saline or nicotine WD. The results showed that among the 71 upregulated genes, the transcription of *Hif3a* was most significantly upregulated (Figure 1A). There are three splice variants of the *Hif3a* gene: the classical HIF‐3α, the NEPAS, and the shortest IPAS [16]. The amino acid sequence between NEPAS and HIF‐3α is similar, differing only in the first exon. The sequence of IPAS is identical to NEPAS, except for the lack of the N‐terminal (Figure 1B). Further transcript type analysis using the transcriptomic data revealed that the most significantly upregulated transcript after nicotine WD was NEPAS/IPAS (Figure 1C). Consistent with this, we designed specific primers to amplify these isoforms and found an increase in the mRNA levels of both NEPAS and IPAS, while the mRNA level of HIF‐3α remained unchanged after WD (Figure 1D–F). Next, we used a monoclonal antibody specifically targeting amino acids 612–639 of HIF‐3α to detect both the classical HIF‐3α and NEPAS but not IPAS due to its truncated structure. Western blotting combined with RT‐qPCR results indicated a significant increase in NEPAS expression after WD (Figure 1G,H), consistent with transcriptomic and qPCR data.

To further explore the potential association of HIF‐3α level with nicotine WD in a human cohort, we analyzed transcriptomic data from postmortem prefrontal cortex samples of current smokers and former smokers (individuals who had undergone smoking cessation). We evaluated the relative mRNA expression levels of HIF‐3α in these two groups. Consistent with our animal findings, the mRNA level of HIF‐3α was higher in former smokers compared to current smokers (Figure S1A,B). This result further supports our finding that the transcriptional level of the HIF‐3α in the mPFC significantly increased after nicotine WD, suggesting a potential link between HIF‐3α and smoking cessation.

Next, we examined cell‐type‐specific expression of NEPAS using immunofluorescence co‐staining. NEPAS signal was widely expressed in various cell types, including neurons (NeuN+), microglia (Iba1+), OPCs (PDGFRα+), and oligodendrocyte‐lineage cells (Olig2+) (Figure 1I; Figure S2A–F). They are also expressed in neurons across various brain regions, such as the hippocampus, amygdala, and agranular cortex (Figure S2G). However, the expression of NEPAS after nicotine WD in the mPFC was predominantly upregulated in neurons (Figure 1I–J; Figure S2A–F), indicating neuron‐specific upregulation.

Given that mPFC neurons receive both excitatory glutamatergic and inhibitory GABAergic innervation, we detected NEPAS expression following nicotine WD in these two neuronal populations using VGLUT1 and VGAT antibodies. Our results showed NEPAS upregulation in both subsets, a finding consistent with glutamatergic innervation from the PVA to the mPFC. mPFC neurons innervated by PVA include projection pyramidal neurons and GABAergic interneurons. Projection pyramidal neurons give rise to local collaterals that synapse onto other mPFC neurons, while GABAergic interneurons (e.g., PV^+^ or SST^+^ subtypes) modulate local network activity through inhibitory connections. Collectively, our data indicate that nicotine WD induces increased NEPAS expression in mPFC neurons, regardless of whether they receive glutamatergic or GABAergic input (Figure S3).

Given the established role of myelin in cognitive function, we assessed myelin formation during nicotine WD using NG2‐CreERT; Tau‐mGFP transgenic mice, in which newly formed myelin sheaths are labeled by membrane‐bound GFP. We observed a significant reduction in mGFP signal in the mPFC after nicotine WD, indicating impaired myelin formation (Figure 1K–M). Together, these results indicate that nicotine WD led to increased neuronal expression of NEPAS but decreased myelin formation in mPFC, which might be involved in cognitive impairment.

### 2.2 Chemogenetic Activation of PVA–mPFC Neural Circuit Rescued NEPAS Expression in mPFC

**FIGURE 2:**
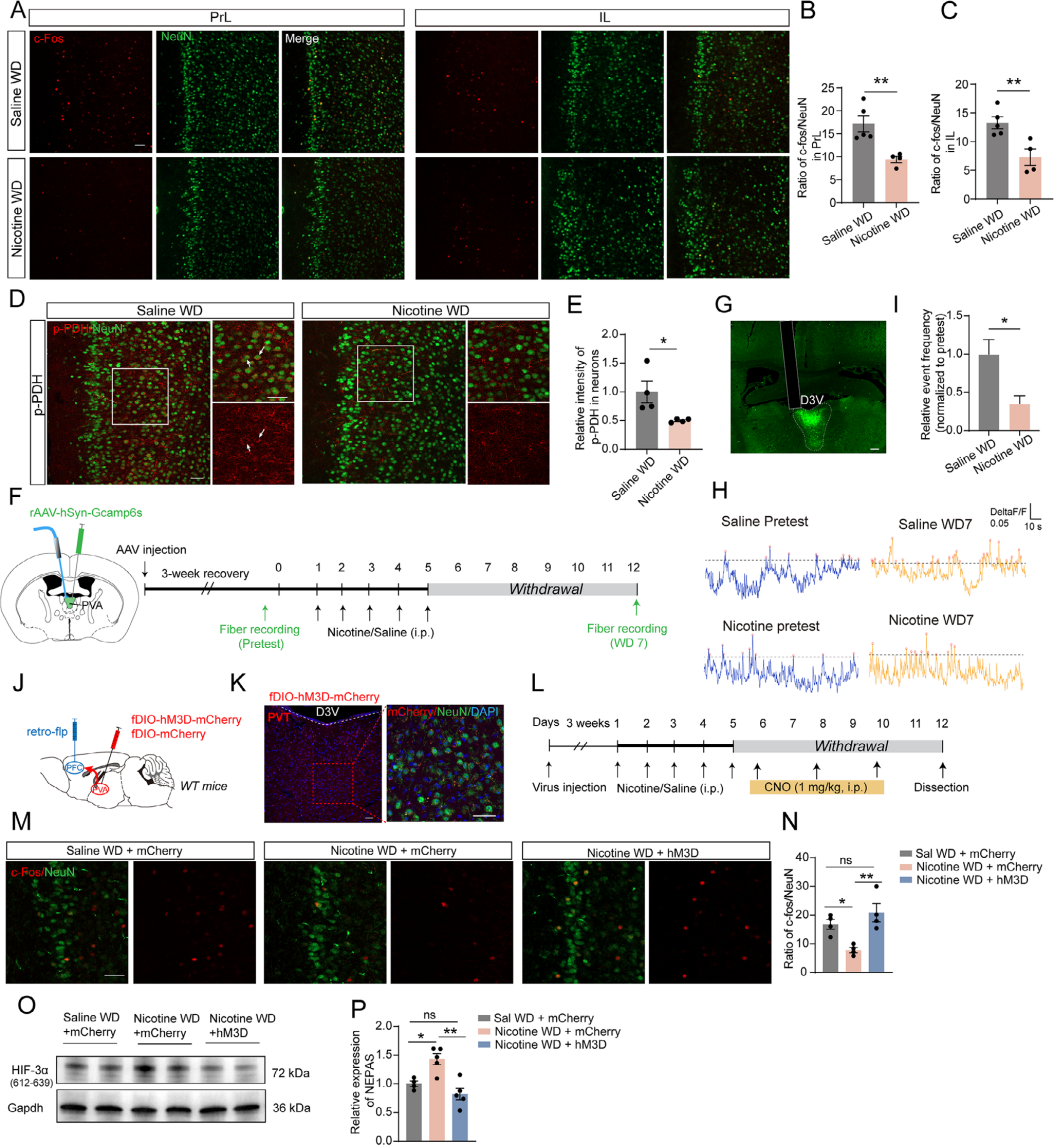
Neuronal activities in the mPFC and PVA were inhibited and chemogenetic activation of PVA–mPFC circuit rescued NEPAS expression. (A) Immunofluorescence images of c‐Fos expression in the mPFC neurons. (B‐C) c‐Fos expression was significantly decreased in both PrL and IL regions after nicotine WD (n = 5, 4 for Saline WD and Nicotine WD, respectively; ^**^ *P* < 0.01, two‐tailed Student's t test). (D) Immunofluorescence images of p‐PDH expression in the mPFC neurons. (E) p‐PDH expression was significantly downregulated in mPFC after nicotine WD (n = 4 for each group; ^*^ *P* < 0.05, two‐tailed Student's t test). (F) Schematic diagram of AAV injection and calcium signal recording procedure. (G) Representative image of PVA after 3 weeks of AAV‐hSyn‐Gcamp6s injection. (H,I) Calcium signals of WD groups were normalized to their pretest signals and nicotine WD significantly decreased Ca^2+^ event frequency in PVA (n = 5, 6 for Saline WD and Nicotine WD, respectively; ^*^ *P* < 0.05, two‐tailed Student's t test). (J) Schematic diagram displaying AAV injection strategies and chemogenetics manipulation procedure during nicotine/saline WD in WT mice. AAV‐hSyn‐flp (retro) was injected into mPFC, and AAV‐hSyn‐fDIO‐hM3D‐mCherry or AAV‐hSyn‐fDIO‐mCherry were injected to PVA. (K) Representative expression of AAV‐hSyn‐fDIO‐hM3D‐mCherry in PVA 3 weeks after injection. (L) The procedure of nicotine WD and CNO was administrated to either groups during withdrawal period. (M‐N) Immunofluorescence staining showed a restored expression of c‐Fos in the mPFC neurons by activating PVA–mPFC circuit (n = 4 for each group, respectively; F (2, 9) = 9.609, *P* = 0.0058; Saline WD + mCherry vs. Nicotine WD + mCherry, ^*^ *P* < 0.05; Nicotine WD + mCherry vs. Nicotine WD + hM3D, ^**^ *P* < 0.01, one‐way ANOVA with Tukey's post hoc test). (O‐P) Immunoblotting of mPFC showed that increased NEPAS expression induced by nicotine WD was restored by activating PVA–mPFC circuit (n = 4, 5, 5 for Saline WD + mCherry, Nicotine WD + mCherry, and Nicotine WD + hM3D, respectively; F (2, 11) = 12.75, *P* = 0.0014; Saline WD + mCherry vs. Nicotine WD + mCherry, ^*^ *P* < 0.05; Nicotine WD + mCherry vs. Nicotine WD + hM3D, ^**^ *P* < 0.01, one‐way ANOVA with Tukey's post hoc test). Scale bars, (I) 200 µm, (A, D, K, and M) 50 µm. Data are presented as mean ± SEM.

Neuronal activity plays a critical role in CNS angiogenesis [11]. First, we assessed neuronal activity after nicotine WD in the mPFC using c‐Fos staining and found a significant decrease in c‐Fos positive neurons, indicating inhibited neuronal activity in the mPFC (Figure 2A–C). Since activity is a crucial regulator of neuronal metabolic level, we also assessed the metabolic status of mPFC neurons following nicotine WD by immunofluorescence staining. We found that nicotine WD did not significantly alter the expression of lactate dehydrogenase A (LDHA) (Figure S4A,B), but significantly reduced the expression of p‐PDH in mPFC neurons (Figure 2D,E), indicating mitochondrial oxidative phosphorylation is enhanced in neurons after nicotine WD.

The PVT region is a critical hub in nicotine WD [31]. Next, we conducted transcriptomic analysis of the PVT following saline or nicotine WD and observed that the transcription levels of several nicotinic acetylcholine receptor genes, including *chrna1, chrna3, and chrna6*, were downregulated after nicotine WD (Figure S5A,B). Among these, the downregulation of *chrna3* was the most significant, as confirmed by RT‐qPCR (Figure S5C). Immunohistochemistry results further showed a decrease in CHRNA3 signal in the anterior of the PVA after nicotine WD (Figure S5D). Given that nicotinic acetylcholine receptors function as calcium channels, we evaluated the cellular calcium levels in PVA neurons. By injecting a hSyn‐Gcamp6s virus into the PVA, we were able to express a calcium sensor in these neurons. After a 3‐week recovery period, we recorded the baseline calcium levels of PVA neurons (Pretest), followed by nicotine treatment and WD (Figure 2F,G). We found that the calcium signals in PVA neurons were significantly decreased after nicotine WD (Figure 2H,I), consistent with the reduced CHRNA3 expression. Additionally, c‐Fos staining also revealed a decrease in the number of activated neurons in both the PVA and the pPVT after nicotine WD (Figure S6A–D). These findings indicated that nicotine WD leads to an inhibition of neuronal activities in the PVA.

Subsequently, we employed chemogenetics to activate mPFC neurons innervated by PVA neurons during nicotine WD, and evaluated the expression of NEPAS in WT mice. A retrograde virus AAV‐hSyn‐flp (retro‐flp) was injected into the mPFC, and a flp‐dependent hSyn‐DIO‐hM3D AAV (fDIO‐hM3D‐mCherry) or its control fDIO‐mCherry was injected into the PVA, thus enabling specific expression of hM3D receptors in PVA neurons that project to the mPFC (Figure 2J). After a 3‐week recovery period, the expression of hM3D‐mCherry/mCherry was confirmed in PVA neurons, appearing as a membrane‐like signal (Figure 2K). Mice were then subjected to a nicotine WD procedure, during which they were treated with Clozapine N‐oxide (CNO) to activate mPFC neurons innervated by PVA neurons (Figure 2L). The c‐Fos staining results showed that nicotine WD induced inhibited c‐Fos expression was efficiently restored by activating PVA–mPFC circuit, indicating neuronal activity in mPFC was rescued (Figure 2M,N). Furthermore, the increased expression of NEPAS in the mPFC was significantly reversed after circuit activation (Figure 2O,P). These results suggest that nicotine WD inhibits neuronal activity in the PVA–mPFC circuit, leading to elevated NEPAS expression in the mPFC.

### 2.3 Neuronal NEPAS Suppresses PTX3 Secretion to Inhibit Angiogenesis

**FIGURE 3:**
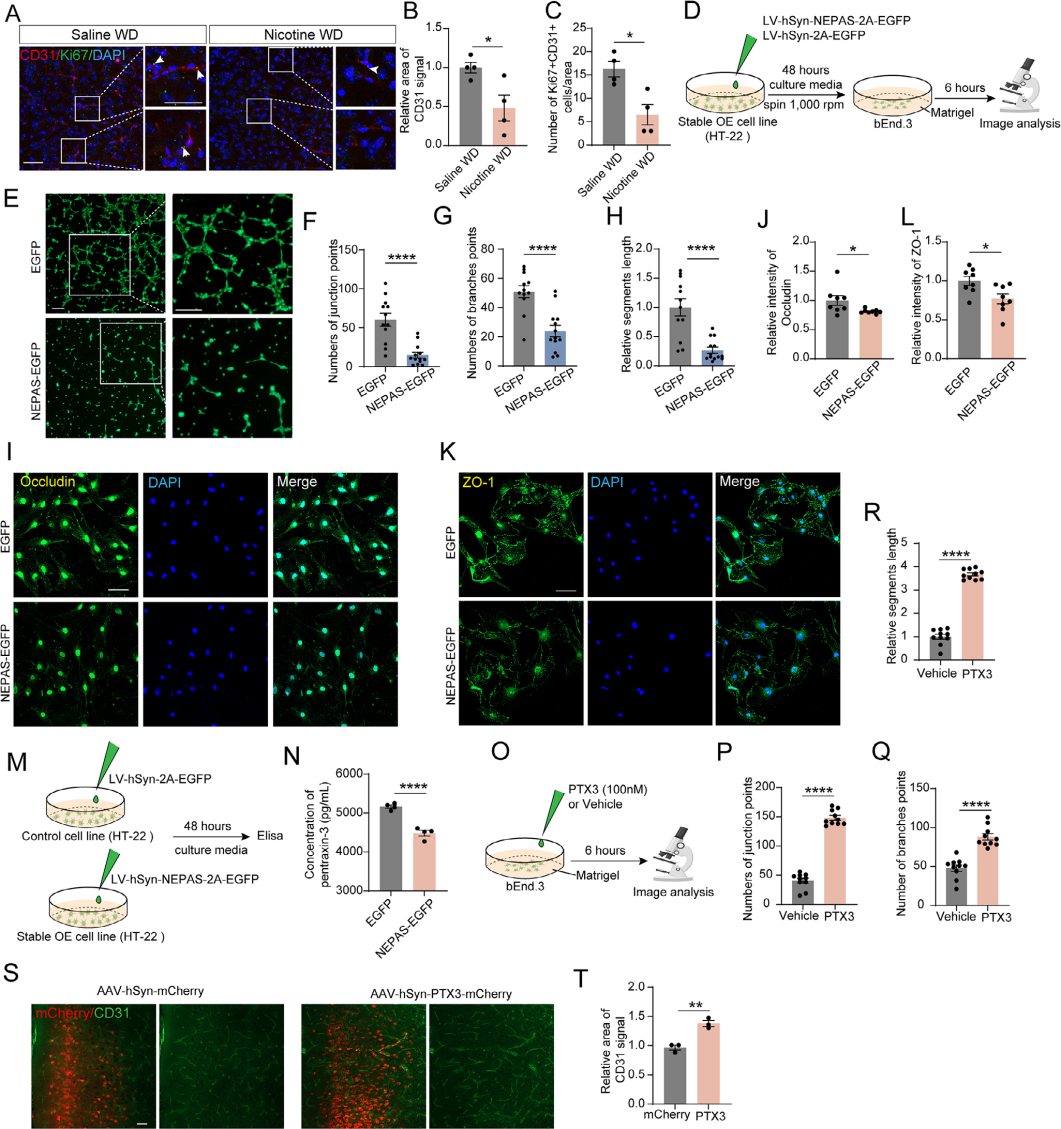
Enhanced expression of neuronal NEPAS inhibited PTX3 releasing and angiogenesis. (A) Representative immunofluorescence images of CD31 and Ki67 co‐staining in the mPFC following saline or nicotine WD. (B) Expression of CD31 in mPFC was decreased after nicotine WD (n = 4 for each group, ^*^ *P* < 0.05, two‐tailed Student's *t*‐test). (C) Co‐localization of CD31 and Ki67 was decreased after nicotine WD (n = 4 for each group, ^*^ *P* < 0.05, two‐tailed Student's t test). (D) Experimental procedure of conditioned media culture of bEnd.3 cells. Stable NEPAS‐expressing (NEPAS‐EGFP) or control (EGFP) HT‐22 cell lines were cultured for 48 h and the culture media was collected to treat bEnd.3 cells seeded in Matrigel‐coated 24‐well plate for 6 h before tube formation evaluation by microscope imaging. (E‐H) Representative images (E) and statistical analysis (F–H) of bEnd.3 cells cultured in vitro showed that treating with media from NEPAS‐EGFP group HT‐22 cells significantly inhibited the tube formation ability of bEnd.3 when compared to EGFP control group, as shown by decreased numbers of junction points, numbers of branches points, and relative segment length (n = 12, 13 for EGFP and NEPAS‐EGFP, respectively; ^****^ *P* < 0.0001, two‐tailed Student's t test). (I,J) Representative image (I) and intensity statistics (J) of bEnd.3 cells showed a decrease in Occludin expression in NEPAS‐EGFP group compared to EGFP group (n = 8 for each group, ^*^ *P* < 0.05, two‐tailed Student's t test). (K‐L) Representative image (K) and intensity statistics (L) of bEnd.3 cells showed a decrease in ZO‐1 expression in NEPAS‐EGFP group compared to EGFP group (n = 8 for each group, ^*^ *P* < 0.05, two‐tailed Student's t test). (M) Control (EGFP) or stable NEPAS‐expressing (NEPAS‐EGFP) HT‐22 cell lines were cultured for 48 h, and the conditioned medium was collected to measure PTX3 levels by ELISA. (N) PTX3 secretion in NEPAS‐EGFP cells was significantly decreased compared to EGFP control cells (n = 4 for each group; ^****^ *P* < 0.0001, two‐tailed Student's t test). (O–R) PTX3 treatment significantly enhanced the tube formation capability of bEnd.3 cells compared to the vehicle group, as shown by increased numbers of junction points, numbers of branches points, and relative segment length (n = 10 for each group, ^****^ *P* < 0.0001, two‐tailed Student's t test). (S) Immunofluorescence images of CD31 signal after overexpression of neuronal PTX3. (T) Overexpression of neuronal PTX3 significantly upregulated CD31 signals in mPFC (n = 3 for each group, ^**^ *P* < 0.01, two‐tailed Student's *t*‐test). Scale bars, 50 µm (A, I, K, S), 200 µm (E). Data are presented as mean ± SEM.

NEPAS has been reported to have the potential to negatively regulate retinal angiogenesis [17]. Consistent with this, we found that nicotine WD caused a significant decrease in CD31 expression, a marker of vascular endothelial cells (Figure 3A,B), as well as a significant reduction in the co‐localization of Ki67 and CD31 in the mPFC (Figure 3C), indicating inhibited angiogenesis following nicotine WD.

To explore the potential role of neuronal NEPAS in angiogenesis formed by microvascular endothelial cells, we stably overexpressed NEPAS in HT‐22 neuronal cells to assess whether its upregulation alters endothelial cell function by culturing bEnd.3 with conditioned medium from transfected HT‐22 cells. Stably NEPAS‐expressing HT‐22 cells were obtained by FACS sorting after 72 h of treating with either NEPAS‐overexpressing lentivirus (NEPAS‐EGFP) or control lentivirus (EGFP). The GFP signal in HT‐22 cells indicated successful construction of stably expressed cell line (Figure S7A,B), and the increased expression of NEPAS was confirmed by RT‐qPCR and western blotting (Figure S7C–E). After 48 h of culture, the culture medium from HT‐22 was collected and used to treat bEnd.3 cells seeded in Matrigel‐coated plate for 6 h (Figure 3D). The angiogenesis function of by bEnd.3 was assessed and we found a significant inhibition of tube formation ability after treating with NEPAS‐overexpressed HT‐22 culture medium compared to the control group, as shown by a significant decrease in number of junction points, number of branch points, and relative segment length (Figure 3E–H).

In addition, we further evaluated the expression of tight junction proteins including Occludin and ZO‐1 in bEnd.3 cultured with HT‐22 media for 48 h. The immunofluorescence staining results showed that expression of either Occludin or ZO‐1 was decreased in bEnd.3 when cultured with media from HT‐22 overexpressed with NEPAS (Figure 3I–L). These results suggested that overexpression of neuronal NEPAS inhibits the angiogenesis ability of microvascular endothelial cells.

To further explore the regulatory mechanism of neuronal NEPAS in angiogenesis, we focused on PTX3, a downstream target gene of NEPAS that encodes a secreted protein reported to mediate angiogenesis [32]. Previous studies have shown that NEPAS negatively regulates PTX3 expression [33]. We first examined the effect of NEPAS overexpression on PTX3 secretion in neuronal cell line cultures and found a significant decrease in PTX3 levels in the culture media of stable NEPAS‐expressing (NEPAS‐EGFP) HT‐22 cells compared to control cell lines (Figure 3M,N). Given that neuronal NEPAS expression is regulated by neuronal activity, we investigated the impact of neuronal activity on PTX3 secretion. On day 5 of primary culture, we transfected hSyn‐hM4D‐mCherry to overexpress hM4D in primary cortical neurons for one week, followed by treatment with CNO to inhibit neuronal activity for 2 days. Culture media were collected and PTX3 levels were measured by ELISA. We observed a dramatic reduction in PTX3 secretion upon neuronal inactivation (Figure S8A,B), indicating that PTX3 secretion from neurons is dependent on neuronal activity.

Next, we assessed the effect of PTX3 on the angiogenic capacity of microvascular endothelial cells. PTX3 enhanced the proliferation rate of bEnd.3 cells (Figure S9A,B), consistent with previous study [34]. Furthermore, PTX3 significantly promoted tube formation by bEnd.3 cells, as evidenced by marked increases in number of junction points, number of branch points, and relative segment length compared to the vehicle group (Figure 3O,R). To validate the effect of neuronal PTX3 on angiogenesis in vivo, we overexpressed PTX3 in mPFC neurons by injecting AAV‐hSyn‐PTX3‐mCherry virus (PTX3), in which PTX3 was expressed together with the mCherry reporter via a linker, or a control AAV‐hSyn‐mCherry virus (mCherry) into the mPFC. After a 3‐week recovery period, the expression level of CD31 in the mPFC was examined. The results showed that, compared with the mCherry group, CD31 expression was significantly increased upon elevation of neuronal PTX3 levels. Moreover, we observed several co‐localizations of PTX3 with CD31 signals, suggesting that neuronal PTX3 promotes angiogenesis (Figure 3S,T). Collectively, these results indicate that PTX3 secretion is dependent on neuronal activity, and overexpression of NEPAS in neurons suppresses PTX3 secretion, thereby inhibiting angiogenesis.

### 2.4 NEPAS Knockdown Rescues Angiogenesis, Myelination, and Cognition

**FIGURE 4:**
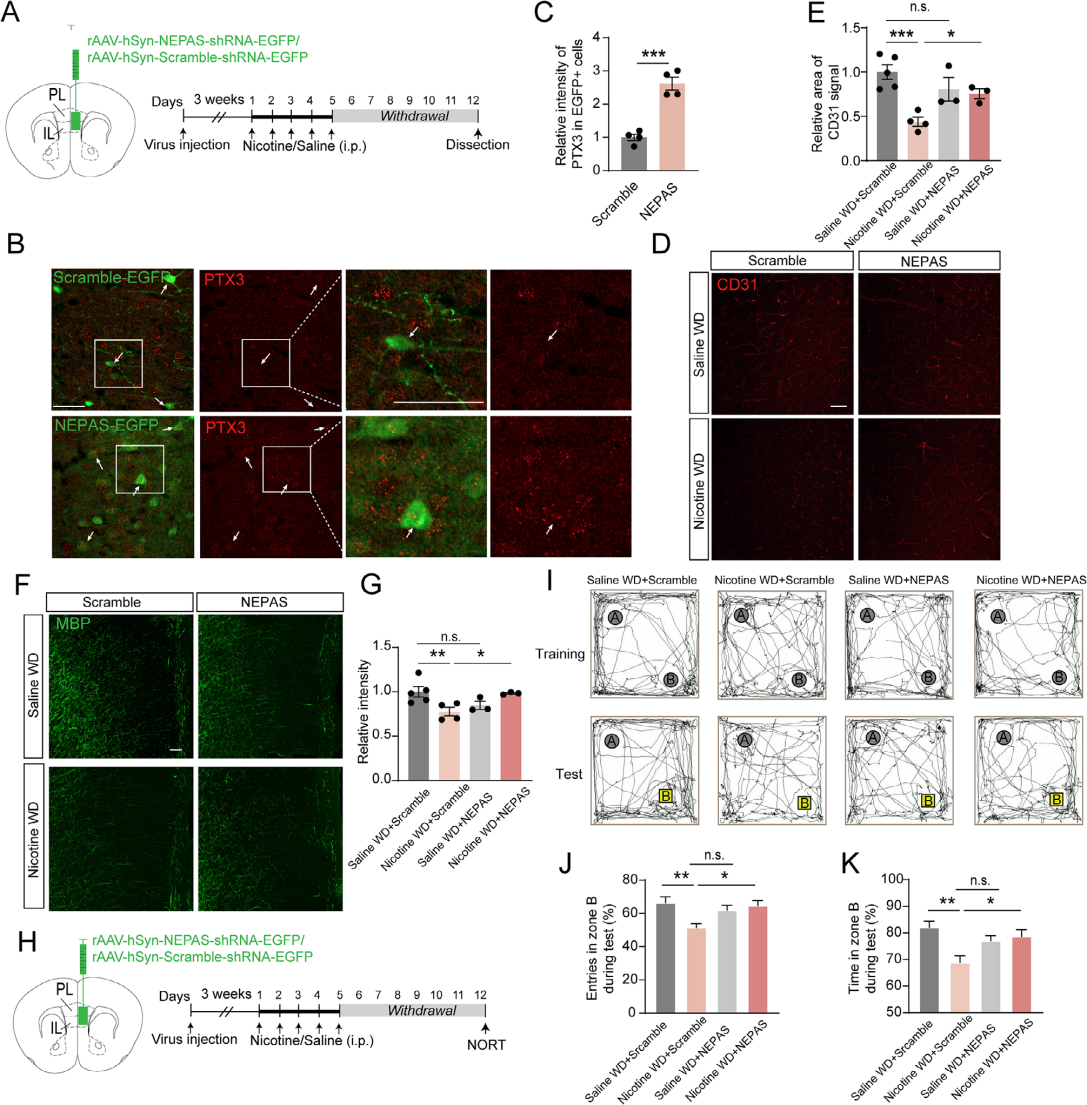
Knockdown of neuronal NEPAS in mPFC enhanced angiogenesis, myelination, and cognitive function. (A) Schematic diagram displaying AAV injection of hSyn‐NEPAS‐shRNA (NEPAS) or control hSyn‐scramble‐shRNA (Scramble) into mPFC. (B) Immunofluorescence images of PTX3 expression in mPFC neurons with or without NEPAS knocking down. (C) Expression of neuronal PTX3 was significantly increased by knocking down NEPAS (n = 4 for each group, ^***^ *P* < 0.001, two‐tailed Student's *t*‐test). (D,E) Nicotine WD induced decreased expression of CD31 was rescued by knockdown neuronal NEPAS in mPFC (n = 5, 4, 3, 3 for Saline WD + Scramble, Nicotine WD + Scramble, Saline WD + NEPAS, and Nicotine WD + NEPAS, respectively; F _withdrawal × shRNA_ (1,11) = 8.905, P = 0.0124; Saline WD + Scramble vs. Nicotine WD + Scramble, ^***^ *P* < 0.001; Nicotine WD + Scramble vs. Nicotine WD + NEPAS, ^*^ *P* < 0.05, two‐way ANOVA with Tukey's post hoc test). (F,G) Nicotine WD induced decreased expression of MBP was restored by knockdown neuronal NEPAS in mPFC (n = 5, 4, 3, 3 for Saline WD + Scramble, Nicotine WD + Scramble, Saline WD + NEPAS, and Nicotine WD + NEPAS, respectively; F _withdrawal × shRNA_ (1,11) = 11.30, *P* = 0.0063; Saline WD + Scramble vs. Nicotine WD + Scramble, ^**^ *P* < 0.01; Nicotine WD + Scramble vs. Nicotine WD + NEPAS, ^*^ *P* < 0.05, two‐way ANOVA with Tukey's post hoc test). (H,I) Schematic diagram of NORT procedure after knockdown of neuronal NEPAS. (J‐K) The impaired NORT memory was restored by knocking down neuronal NEPAS, as shown by increased entries (J) (n = 12, 12, 11, 11 for Saline WD + Scramble, Nicotine WD + Scramble, Saline WD + NEPAS, and Nicotine WD + NEPAS, respectively; F _withdrawal × shRNA_ (3,42) = 4.892, *P* = 0.0053; Saline WD + Scramble vs. Nicotine WD + Scramble, ^**^ *P* < 0.01; Nicotine WD + Scramble vs. Nicotine WD + NEPAS, ^*^ *P* < 0.05, two‐way ANOVA with Tukey's post hoc test) and exploration time to the novel object (K) (n = 12, 12, 11, 11 for Saline WD + Scramble, Nicotine WD + Scramble, Saline WD + NEPAS, and Nicotine WD + NEPAS, respectively; F _withdrawal × shRNA_ (3,42) = 5.637, *P* = 0.0024; Saline WD + Scramble vs. Nicotine WD + Scramble, ^**^ *P* < 0.01; Nicotine WD + Scramble vs. Nicotine WD + NEPAS, ^*^ *P* < 0.05, two‐way ANOVA with Tukey's post hoc test). Scale bars, 50 µm. Data are presented as mean ± SEM.

Next, we used an AAV carried shRNA to knock down NEPAS in neurons during nicotine WD and evaluated the angiogenesis in mPFC (Figure 4A). Mice were bilaterally injected with AAV‐hSyn‐NEPAS‐shRNA or control AAV‐hSyn‐scramble‐shRNA virus in mPFC, and after a 3‐week recovery, the expression of NEPAS in neurons shown by immunofluorescence was significantly reduced in the mPFC (Figure S10A,B). Interestingly, PTX3 levels in NEPAS‐knockdown neurons were significantly increased (Figure 4B,C), consistent with the decreased levels observed in NEPAS‐overexpressing HT‐22 cells in vitro as shown in Figure 3L,M. In addition, we found that nicotine WD caused decreased CD31 expression was efficiently reversed by knocking down neuronal NEPAS (Figure 4D,E), suggesting that neuronal NEPAS may negatively regulate angiogenesis.

We also evaluated the effect of neuronal NEPAS knockdown on myelin level in mPFC. We found that knock down of neuronal NEPAS in mPFC did not affect the expression of MBP, however, the decreased level of MBP following nicotine WD was significantly restored (Figure 4F,G), suggesting that increased neuronal expression of NEPAS by nicotine WD inhibits myelination.

To further examine the effect of neuronal NEPAS in nicotine WD induced cognitive deficits, mice were bilaterally injected with AAV‐hSyn‐NEPAS‐shRNA or control virus in mPFC. After a 3‐week recovery, they underwent the nicotine WD procedure followed by the NORT task (Figure 4H,I). Knocking down of neuronal NEPAS did not affect the learning ability in NORT task (Figure S11A–C), however, the impaired cognitive memory caused by nicotine WD was reversed by knocking down of NEPAS in mPFC neurons, as shown by restored entries (Figure 4J) and exploring times (Figure 4K) to the zone of novel object in Nicotine + Scramble group compared to Nicotine + NEPAS group. Together, these results indicate that mPFC neuronal NEPAS inhibits angiogenesis, decreased myelin level, and causes cognitive deficits after nicotine WD.

### 2.5 Activating PVA–mPFC Circuit During WD Restores Neurovascular and Cognitive Function

**FIGURE 5:**
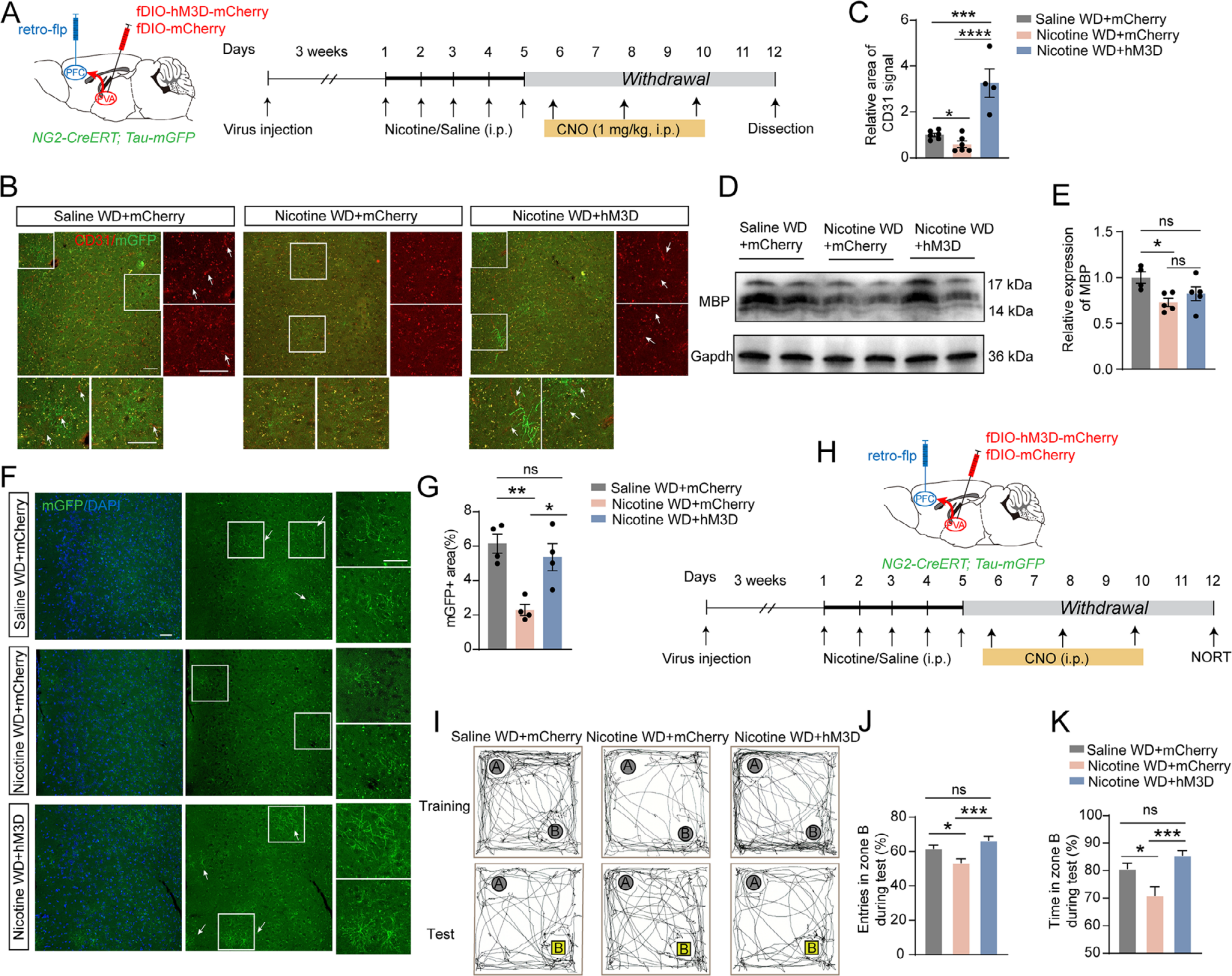
Chemogenetic activation of PVA–mPFC circuit rescued mPFC neurovascular and cognitive function. (A) Schematic diagram displaying chemogenetics AAV injection strategies manipulation procedure during nicotine/saline WD in NG2‐CreERT; Tau‐mGFP mice to label newly formed myelin sheath. (B,C) Representative images of CD31 expression in mPFC showed inhibited expression of CD31 caused by nicotine WD was rescued after activating PVA–mPFC circuit (n = 6, 6, 4 for Sal WD + mCherry, Nic WD + mCherry, and Nic WD + hM3D, respectively; F (2,13) = 22.80, ^****^ *P* < 0.0001; Sal WD + mCherry vs. Nic WD + mCherry, ^*^ *P* < 0.05; Sal WD + mCherry vs. Nic WD + hM3D, ^***^ *P* < 0.001; Nic WD + mCherry vs. Nic WD + hM3D, ^****^ *P* < 0.0001, one‐way ANOVA with Tukey's post hoc test). (D,E) Immunoblotting of mPFC showed that decreased MBP induced by nicotine WD were not significantly affected by activating mPFC neurons innervated by PVA neurons (n = 4, 5, 5 for Saline WD + mCherry, Nicotine WD + mCherry, and Nicotine WD + hM3D, respectively; F (2,11) = 4.505, *P* = 0.0372; Saline WD + mCherry vs. Nicotine WD + mCherry, ^*^ *P* < 0.05, one‐way ANOVA with Tukey's post hoc test). (F,G) Representative images of mGFP expression in mPFC showed decreased myelin formation caused by nicotine WD was rescued after activating mPFC ‐PVA circuit (n = 4 for each group, F (2,9) = 11.98, *P* = 0.0029; Saline WD + mCherry vs. Nicotine WD + mCherry, ^**^ *P* < 0.01, Nicotine WD + mCherry vs. Nicotine WD + hM3D, ^*^ *P* < 0.05, one‐way ANOVA with Tukey's post hoc test). (H,I) Schematic diagram of NORT procedure for chemogenetics activating mPFC‐PVA neural circuit. (J) The impaired NORT memory was restored by activating this circuit, as shown by increased entries (J) (n = 11 for each group, F (2,30) = 9.191, *P* = 0.0008; Saline WD + mCherry vs. Nicotine WD + mCherry, ^*^ *P* < 0.05; Nicotine WD + mCherry vs. Nicotine WD + hM3D, ^***^ *P* < 0.001, one‐way ANOVA with Tukey's post hoc test) and exploration time to zone of novel object (K) (n = 11 for each group, F (2,30) = 9.980, *P* = 0.0005; Saline WD + mCherry vs. Nicotine WD + mCherry, ^*^ *P* < 0.05; Nicotine WD + mCherry vs. Nicotine WD + hM3D, ^***^ *P* < 0.001, one‐way ANOVA with Tukey's post hoc test). Scale bars, 50 µm. Data are presented as mean ± SEM.

As neuronal NEPAS expression is regulated by the activity of PVA–mPFC circuit, we further examined the effect of this circuit on angiogenesis and cognitive function. A retrograde virus AAV‐hSyn‐flp (retro‐flp) was injected into the mPFC, and a flp‐dependent hSyn‐DIO‐hM3D AAV (fDIO‐hM3D‐mCherry) or its control fDIO‐mCherry was injected into the PVA. After a 3‐week recovery period, mice were then subjected to a nicotine WD procedure, during which they were treated with Clozapine N‐oxide (CNO) to activate mPFC neurons innervated by PVA neurons (Figure 5A). We observed that, the angiogenesis was significantly restored by activating this neural circuit, as shown by the increased expression of CD31 in the Nicotine WD + hM3D group compared with Nicotine WD + mCherry group (Figure 5B,C).

Next, we applied this chemogenetic activation in NG2‐CreERT; Tau‐mGFP transgenic mice to evaluate the myelin formation ability. We found that activation of this neural circuit could not significantly restore total MBP level though with a mild upregulation (Figure 5D,E). Interestingly, the impaired myelin formation caused by nicotine WD in the mPFC was effectively restored by activating this neural circuit (Figure 5F,G).

Finally, we evaluated the regulation of PVA–mPFC circuit on cognitive memory in WT mice (Figure 5H). We found that activating this neural circuit significantly rescued the NORT memory deficits (Figure 5I–K), as shown by a significant increase in both exploration entries (Figure 5J) and times to novel object in Nicotine WD + hM3D group compared to Nicotine WD + mCherry group (Figure 5K), while manipulating this circuit did not affect the learning ability in NORT task (Figure S12A–C). Together, these results indicated that activating PVA–mPFC neural circuit during nicotine WD efficiently rescues neuronal NEPAS mediated angiogenesis, thereby promoting myelin formation and improving cognitive memory.

## 3 Discussion

**FIGURE 6:**
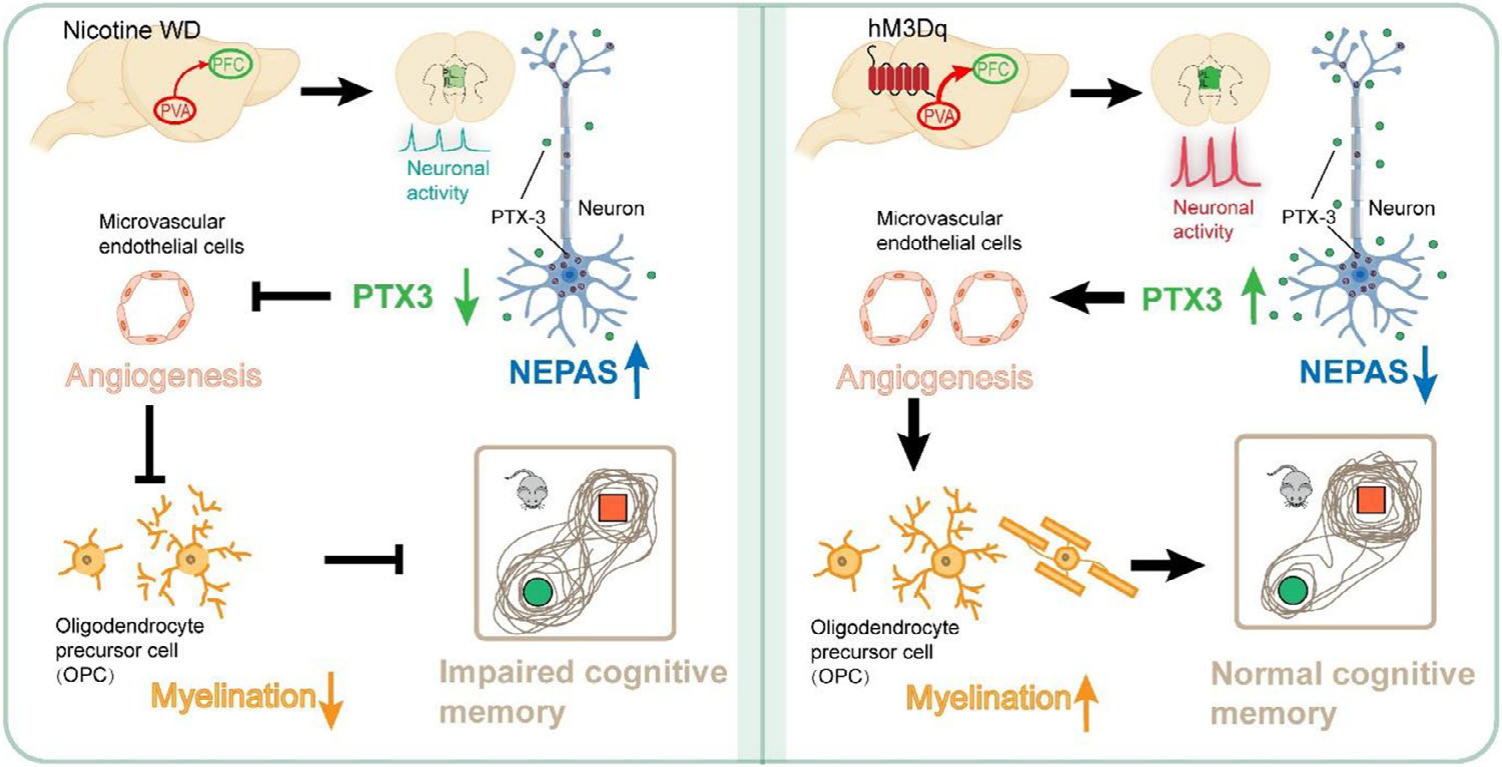
Schematic illustration of the neuron‐derived signaling axis in angiogenesis and their role in myelination and cognitive function. Left: During nicotine WD, reduced activity in the PVA–mPFC neural circuit leads to upregulation of neuronal NEPAS in the mPFC. Elevated NEPAS suppresses the secretion of PTX3, impairing angiogenesis, resulting in hypomyelination and cognitive memory deficits. Right: Chemogenetic activation of PVA‐innervated mPFC neurons downregulates NEPAS expression, restores angiogenesis, rescues myelination deficits, and improves cognitive function. This schematic illustrates an activity‐dependent neuron‐vascular signaling axis mediated by NEPAS–PTX3 that regulates cognitive outcomes in acquired cognitive impairments.

Our study identifies neuronal NEPAS, an activity‐regulated isoform of HIF‐3α in the mPFC, acts as a critical suppressor of angiogenesis and cognitive memory during nicotine WD. We demonstrate that reduced activity in the PVA–mPFC neural circuit upregulates NEPAS expression in mPFC neurons, which in turn inhibits the secretion of PTX3, leading to impaired angiogenesis, hypomyelination, and cognitive deficits. Conversely, chemogenetic activation of this pathway downregulates NEPAS, restores angiogenesis, and rescues both myelin integrity and memory function (Figure 6). These findings reveal a previously unknown activity‐dependent neuron–vascular signaling axis that links neural circuit hypoactivity to cognitive impairment via transcriptional regulation of angiogenesis.

### 3.1 Critical Role of Angiogenesis in Cognitive Function

Growing evidence suggests that aberrant angiogenesis perturbation plays a critical role in the pathogenesis of types of cognitive disorders, including aging related dementia [9] and AD [10]. Aging is a dominant risk factor for impaired angiogenesis in the elderly [9], which highly contributes to the impaired cognitive function in aged [35]. Notably, vascular contributions to cognitive impairment and dementia (VCID) are one of the leading causes of dementia along with AD [36]. In the brain of AD, cerebral microvasculature is reduced around Aβ plaques, and endothelial cells express the molecular signature of non‐productive angiogenesis and accumulate IB4 reactive vascular anomalies around Aβ plaques, indicating a failure of angiogenesis [37]. Studies from AD human revealed that tau pathology plays a critical role in pathological angiogenesis, contributing to cerebrovascular lesions and neurodegeneration in AD [38]. Animal studies show that angiogenesis inhibitor administration exhibit retarded Morris water maze acquisition [39], while promoting angiogenesis of hippocampus by moxibustion therapy improved delayed memory deficits in a vascular dementia rat model [40]. These studies indicate that interventions to target the angiogenesis process could have beneficial effects on improving cognitive functions in acquired cognitive impairments.

Dysregulation or disruption of myelination can lead to impaired cognitive abilities and is a contributing factor in various neurodegenerative diseases [41, 42]. In order to differentiate and form multiple myelin segments, OPCs have exceptionally high metabolic demands [43]. In fact, angiogenesis‐mediated vascular networks not only supply oxygen and nutrients critical for the high‐energy demands of myelination, but also secrete growth factors and extracellular matrix components that directly regulate OPCs proliferation, differentiation, and myelin formation [44, 45, 46]. In a clinical study, angiogenesis activity was found to be correlated with increased white matter hyperintensities [47]. These studies indicate that sufficient angiogenesis plays a pivotal role in orchestrating myelination, a finding supported by our current study. Consistently, we demonstrate that either directly knocking down neuronal NEPAS (Figure 4D–G) or chemogenetically activating mPFC neurons innervated by PVA neurons (Figure 5B,C,F,G) to restore NEPAS expression is sufficient to enhance angiogenesis, thereby promoting myelin formation, revealing a role for neuronal NEPAS in regulating myelin formation through the modulation of angiogenesis. Notably, newly formed myelin (i.e., myelin formation) constitutes only a small fraction of the total myelin, and MBP blotting reflects overall myelin levels, changes in newly formed myelin are unlikely to significantly affect total MBP levels. Therefore, we observed changes in mGFP signal (Figure 5F,G), but no significant alteration in MBP levels after manipulating the PVA–mPFC circuit (Figure 5D,E).

### 3.2 Neuron‐Derived Signals in the Regulation of Angiogenesis

Angiogenesis is significantly regulated by neuron‐derived signals, such as the Slit‐Robo signaling pathway and the reelin‐ApoER2‐Dab1 signaling cascade in the retina [48, 49]. In the brain, neuron sreated VEGF‐A promotes neovascularization by binding to receptors on endothelial cells [11, 50].

Recently, the regulatory role of neuronal activity in angiogenesis has received widespread attention while the underlying mechanism remains largely unexplored. For example, Christina Whiteus and colleagues demonstrated that repetitive sensorimotor stimuli induce near‐complete arrest of angiogenesis in the corresponding cortical regions. They showed that neuronal hyperactivity suppresses angiogenesis by reducing endothelial cell proliferation and vessel sprouting [51]. In another study, the J. L. Goldberg group found that inhibition of spontaneous cholinergic activity prevents endothelial cell invasion into the deep retinal layers and leads to blood‐retinal barrier (BRB) dysfunction in mice. This activity blockade downregulates VEGF, a major driver of angiogenesis, and Norrin, a Wnt ligand essential for BRB integrity [52]. More recently, S. Biswas and colleagues reported that glutamatergic neuronal activity drives angiogenesis in the retinal deep vascular plexus and induces Norrin expression in Müller glia and interneurons [13]. These compelling studies highlight the growing recognition of neuronal activity as a critical regulator of cerebral and retinal angiogenesis, however, the underlying mechanisms by which neuronal activity influences angiogenesis remain unclear. Our current study addresses this issue, revealing the regulatory effect of PVA–mPFC circuit neuronal activity on angiogenesis and demonstrating that the NEPAS‐PTX3 axis is a crucial molecular mechanism mediating this process.

Neurotransmitter release and synaptic plasticity are both outcomes of neuronal activity. In fact, intrinsic transcriptional responses within neurons occur earlier and play a pivotal role in regulating these processes. For instance, the immediate‐early transcription factor Zif268 is rapidly induced by calcium influx, subsequently enhancing the expression of synaptic vesicle components, thereby increasing the efficiency of vesicle docking and exocytosis [53, 54]. In the context of VEGFA, its expression is controlled by multiple transcription factors, including HIF‐1α, STAT3, and c‐Fos [55]. These findings suggest that transcription factors may serve as upstream molecular switches that regulate the sustained functional and structural adaptations, such as neurotransmitter release and synaptic plasticity, that we observe.

### 3.3 Transcription Factor NEPAS in Neuron Acts as an Upstream Regulator for PTX3 to Modulate Angiogenesis in the Brain

Angiogenesis is tightly regulated by oxygen availability. The HIF family of transcription factors, particularly HIF‐1α, HIF‐2α, and HIF‐3α, serves as the master regulators of this process [56]. Under hypoxic conditions, HIFs stabilize, translocate to the nucleus, and activate genes critical for vascular adaptation [57]. HIF‐1α and HIF‐2α are rapidly stabilized in response to acute hypoxia and promote angiogenesis through multiple mechanisms including VEGF or Notch signaling, et al. [58]. Unlike HIF‐1α and HIF‐2α, HIF‐3α acts as an endogenous inhibitor. It was reported to suppress angiogenesis through a competitive inhibition that HIF‐3α forms non‐functional heterodimers with HIF‐β, thereby blocking HIF‐1α/2α activity [17].

Our current study expanded our understanding on the biological effect of HIF‐3α, especially the NEPAS isoform in neurons. On one hand, we revealed that neuronal expression of NEPAS negatively regulates functions of endothelial cells, as well as the angiogenesis process (Figures 3D–L and 4D,E). On the other hand, we demonstrated that enhancing activities of mPFC neurons innervated by PVA neurons sufficiently restores the expression of NEPAS in the mPFC, thereby enhance angiogenesis and promote myelin formation (Figures 2 and 5), suggesting that neuronal activity triggered neuronal NEPAS is involved in the regulation of myelin formation through affecting angiogenesis.

As a transcription factor, HIF‐3α can regulate the expression of multiple downstream target genes, including PTX3. Previous studies have demonstrated that HIF‐3α exerts a negative regulatory effect on PTX3 expression, consistent with our current findings showing that NEPAS overexpression significantly reduces PTX3 secretion in neurons (Figure 3M,N), whereas NEPAS knockdown enhances PTX3 expression in neurons (Figure 4B,C). Although PTX3 has been reported to play a critical role in angiogenesis regulation after stroke [32, 34], whether PTX3 participates in NEPAS‐mediated regulation of angiogenesis remains unclear. Our in vitro and in vivo results both suggest that PTX3 may act as a downstream effector through which neuronal NEPAS influences vascular formation (Figures 3M–T and 4B–E). Collectively, these findings indicate that NEPAS expression in neurons is dependent on neuronal activity within the PVA–mPFC circuit, and that its regulation of angiogenesis is mediated by modulating PTX3 secretion from neurons. However, it remains unclear how PTX3 influences the function of endothelial cells to form vascular, particularly its molecular target. Although VEGFR2 has been previously implicated in vascular regulation and associated with PTX3 deficiency in earlier studies, whether VEGFR2 is a direct target of PTX3 or whether other targets exist requires further experimental validation in future studies.

### 3.4 Current Strategies and Limitation in Promoting Angiogenesis in the CNS

Angiogenesis is an important protective mechanism that promotes neural regeneration and neuroplasticity to recover from neurological diseases. One of the strategies to promote angiogenesis is targeted on the vascular endothelial growth factor (VEGF) signaling. Zhang et al. found that administration of rhVEGF165 at the late ischemic stage enhances angiogenesis and significantly improves neurological functional recovery [59]. However, administration in the early stage of ischemic increased BBB leakage and disruption of the BBB integrity [60], though it could be avoided by combined use of Ang‐1 to protect damaged cells without affecting BBB permeability [61]. Other potential strategies to promoting angiogenesis includes activating HIF‐related signaling [62] and cell‐based therapies [63]. However, these studies are still experimental, and further clinical applications are need to applied to validate. Our current study reveals a critical role of neuronal NEPAS in regulating brain angiogenesis, highlighting its potential as a therapeutic target for promoting angiogenesis and treating acquired cognitive impairments.

### 3.5 Limitations of the Current Study

The shRNA used targets a shared sequence between NEPAS and IPAS due to high sequence homology. However, as NEPAS, but not IPAS, is significantly upregulated after nicotine WD, and IPAS expression remains low and unchanged, we attribute the rescue effects primarily to NEPAS knockdown. A potential contribution of IPAS cannot be fully excluded, but its lack of regulation in this model suggests a minimal role.

Although we have demonstrated that NEPAS expression is upregulated in mPFC neurons following nicotine WD and that chemogenetic activation of the PVA–mPFC circuit restores its expression, the specific signaling pathways linking inputs from the PVA–mPFC circuit to NEPAS transcription remain to be elucidated. Our results show increased expression of neuronal p‐PDH (Figure 2D,E), indicating that nicotine WD remodels mitochondrial metabolism in mPFC neurons. This metabolic reprogramming may interact with intracellular components, ultimately leading to altered NEPAS expression. Furthermore, chemogenetic manipulation of the PVA–mPFC circuit may modulate neuronal metabolic states by affecting neuronal activity, thereby inducing changes in NEPAS expression. However, the precise mechanisms by which altered neuronal activity regulates NEPAS expression require further investigation.

In addition, it is noteworthy that gender is a key biological variable influencing observed drug withdrawal effects and neurovascular responses [64, 65, 66]. The primary reasons for selecting male mice in this study are as follows: (1) the majority of current research on the impact of drug withdrawal on cognitive and memory functions employs male rodents, which facilitates direct comparison with existing literature; (2) the estrous cycle in female mice induces fluctuations in hormone levels, and such physiological variations may interfere with measurements of neurovascular coupling, metabolic states, and behavioral performance. Utilizing male mice helps minimize the influence of these confounding factors. Nevertheless, we acknowledge that the effects observed in male mice may not necessarily be generalizable to females, and thus future studies should extend these experiments to female subjects to explore potential sex‐specific mechanisms.

Notably, analysis of human prefrontal cortex transcriptomic data reveals that HIF‐3α transcription is elevated in an individual who underwent smoking cessation compared to two control individuals with ongoing tobacco use (Figure S1). It should be noted that the difference between former smoker and current smokers may be influenced by other potential confounders except for nicotine WD, such as age, duration and intensity of smoking, cessation time, and comorbid psychiatric conditions. The elevated HIF‐3α observed in former smokers compared to current smokers may reflect not only nicotine WD but also broader behavioral or physiological adaptations associated with smoking cessation. Therefore, these human cohort data provide correlational support for the involvement of HIF‐3α in the smoking cessation process, but do not establish a direct causal relationship. Our animal models, in which nicotine dosage is strictly controlled, offer mechanistic insights that help explain this phenomenon and complement these human observations. Although preliminary and limited by sample size, this observation suggests that HIF‐3α pathway dysregulation may be conserved in humans and warrants further investigation in larger, well‐characterized cohorts. Future studies in larger cohorts are needed to validate NEPAS and PTX3 alterations in individuals with tobacco use disorder.

## 4 Conclusion

Collectively, our study demonstrates that nicotine WD induces hypoactivity in the PVA–mPFC neural circuit, leading to upregulation of NEPAS in mPFC neurons. Elevated neuronal NEPAS suppresses the activity‐dependent secretion of PTX3, impairing angiogenesis, disrupting myelination, and ultimately causing cognitive deficits. Chemogenetic activation of the PVA–mPFC circuit rescues NEPAS expression, restores neurovascular integrity, improves myelination and reverses cognitive impairment. Notably, analysis of human postmortem prefrontal cortex data reveals elevated HIF‐3α expression in former smokers, supporting the translational relevance of this pathway. We identify an activity‐dependent NEPAS–PTX3 axis in mPFC neurons that couples neural circuit hypoactivity to impaired neurovascular coupling and cognitive dysfunction. This pathway not only advances our understanding of how neural circuits regulate brain homeostasis but also highlights the PVA–mPFC circuit and its downstream NEPAS–PTX3 signaling axis as potential targets for neuromodulation‐based interventions in cognitive disorders associated with prefrontal hypoactivity.

## 5 Experimental Section/Methods

### 5.1 Animals

Adult male Wild type C57BL/6 J mice (8–10 weeks) were purchased from Beijing Vital River Laboratory Animal Technology company. NG2‐CreERT; Tau‐mGFP mice were bred on C57BL/6 J background. All animal procedures were approved by the Laboratory Animal Ethics Committee of Shantou University Medical College (Animal Use Protocol #SUMC2025‐007) and conducted in accordance with the National Institutes of Health Guide for the Care and Use of Laboratory Animals. Mice were housed under standard conditions of a reversed 12‐h light/dark cycle with access to food and water ad libitum, and behavioral experiments were carried out during the dark cycle. All animals were randomly assigned using a blocked design that accounted for litter and age, ensuring that mice from the same litter were distributed across different experimental groups. This approach minimized potential confounding effects due to shared genetic background or housing conditions.

### 5.2 Drug and Reagent

(‐)‐Nicotine hydrogen tartrate (SML1236, Sigma‐Aldrich) was dissolved in sterile 0.9% saline, and the pH was adjusted to 7.4 using a 1 M sodium hydroxide solution. The nicotine solution was then sterilized by filtration through a 0.22‐µm syringe filter and administered intraperitoneally (i.p.) at a dosage of 1.5 mg/kg. Tamoxifen (T5648, Sigma‐Aldrich) was dissolved in corn oil at 25 mg/ml and administered (i.g.) at a dosage of 100 mg/kg. Clozapine N‐oxide (C0832, Sigma‐Aldrich) was dissolved in saline and administered (i.p.) at a dosage of 1 mg/kg on days 1, 3, and 5 during WD. The full‐length mature mouse pentraxin‐3 protein (HY‐P71246, MedChemExpress) was dissolved in DMEM containing 10% FBS and applied to cells at a concentration of 100 nM. Control groups received an equivalent volume of the vehicle.

### 5.3 Immunofluorescence and Immunohistochemistry Staining

Mice were anesthetized with isoflurane (3.5% induction, 1.5–2% maintenance, RWD, China) and transcardially perfused with 0.9% saline followed by 4.0% paraformaldehyde in 0.1 M PBS (pH 7.4). The entire brain was quickly removed, processed for post‐fixing in 4.0% paraformaldehyde for 4 h, and cryoprotected with 30% sucrose/phosphate‐buffered saline (PBS) for 3 days. The brain was sectioned into 30‐µm slices by a vibratome (Leica). After blocking in 5.0% normal goat serum in PBS, floating sections were incubated in primary antibody (c‐Fos, Synaptic Systems, 226008; HIF‐3α, 27650‐1‐AP, Proteintech; CD31, 550274, BD Biosciences; NeuN, MAB377, Millipore; Ki67, HA720163F, HUABIO; Olig2, HA601558, HUABIO; Iba1, ab5076, Abcam; PDGFRα, AF1062‐SP, R&D Systems; LDHA, 19987‐1‐AP, Proteintech; p‐PDH, 37115, Cell Signaling Technology; PTX3, 13797‐1‐AP, Proteintech and sc‐373951, Santa Cruz Biotechnology; VGLUT1, HA601392, HUABIO; VGAT, HA601384, HUABIO) at 4°C overnight. After rinsing in PBS, sections were incubated for 1 h with the Alexa Fluor cy3‐, 488‐ or 594‐conjugated secondary antibodies (Jackson ImmunoResearch). 4′, 6‐Diamidino‐2‐phenylindole (DAPI) (1:106, CST, #4083) was used to stain the nuclei. The sections were then washed and visualized under an LSM 800 laser confocal fluorescence microscope (Carl Zeiss).

The brain slices were also analyzed with immunohistochemistry by blocking endogenous peroxidase with endogenous peroxidase blocking buffer (P0100B, Beyotime Biotechnology.) and followed by incubating with primary antibodies [CHRNA3, 10333‐1‐AP, Proteintech] at 4°C overnight. After rinsing in PBS, sections were incubated for 1.5 h with the horseradish peroxidase (HRP)‐conjugated anti‐rabbit IgG. Sections were then rinsed and incubated at room temperature for 15 min in visualization buffer containing 3,3 N‐Diaminobenzidine Tetrahydrochloride (DAB). The sections were then washed and visualized by an image system (Zeiss A2).

### 5.4 RNA‐Sequencing and Data Processing

The mPFC and PVA tissues of mice were dissected under a microscope using stereotactic coordinates with a mouse matrix (Plastic One Inc.). After nicotine or saline WD procedure, the total RNA was extracted using ice‐cold TRIZOL reagent (15596026, ThermoFisher, Waltham, MA, USA). The RNA Nano 6000 Assay Kit of the Bioanalyzer 2100 system (Agilent Technologies, Santa Clara, CA, USA) was used to evaluate RNA integrity numbers (RINs) and concentrations. Only samples with RIN > 7.0 were used to conduct library preparation and RNA sequencing process. The RNA‐seq library was prepared and sequenced on the Illumina xplus platform and 150 bp paired‐end reads were generated (Beijing SeqWisdom Biotechnology Co., Ltd., Beijing 100176, China.). Read quality was assessed with FastQC, and reads were aligned to the mouse genome (mm10) using histat2 with default parameters. Aligned reads were subjected to Featurecount for read counting. The different expressed genes (DEGs) analysis in RNA sequencing data was compared between nicotine withdrawal and saline withdrawal by using DESeq2, and the criteria were *P* < 0.05, log_2_ fold change > 0.585, or log_2_ fold change < – 0.585.

Human transcriptomic data were obtained from the Gene Expression Omnibus (GEO, accession number GSE182173). This dataset originated from a study investigating the impact of alcohol consumption on cortical function, in which the experimental group consisted of individuals with a history of alcohol intake and the control group included those without such a history, with both groups further comprising active tobacco use (current smoker) and smoking cessation (former smoker). To eliminate potential confounding effects of alcohol consumption, we exclusively utilized data from the control group – individuals without a history of alcohol intake, to analyze the expression level of HIF‐3α in current and former smokers. Prefrontal cortex samples from the former smoker and current smokers were analyzed by analyzing the counts per million (CPM) of HIF‐3α.

### 5.5 RNA Extraction, Reverse Transcription, and Real‐Time PCR

Total RNA was extracted after 48 h of nicotine treatment by RNA extraction reagent according to the manufacture instruction (Vazyme). The reverse transcription with random primers was conducted according to the HiScript III All‐in‐one RT SuperMix reagent (Vazyme). Real‐time polymerase chain reaction (PCR) amplification of the complementary DNA was performed on samples in triplicate with SYBR qPCR Master Mix (Vazyme). Relative mRNA expression was normalized to the internal control Actb. The primers (5′‐3′) for examining genes transcription level were shown in Table 1.

**TABLE 1:** Primer sequences of RT‐qPCR.

Genes	Forward (5’‐3’)	Reverse (5’‐3’)
*Actb*	Ggtcatcactatcggcaat	gtgttggcatagaggtctt
*Hif3α* (F1R1)	Ttggggctgcagcgcgtgag	cggtgcatgcgcaggtagctg
*Hif3α* (F2R2)	Gaagttcacatactgcgacga	gtccaaagcgtggatgtattcat
*Hif3α* (F3R3)	Atggactgggaccaagacagg	tgctgcgcagaggcggtgcat
*Chrna3*	Ggtcatcactatcggcaat	gtgttggcatagaggtctt

### 5.6 Brain Lysate Preparation and Western Blotting

The mPFC tissues were dissected under a microscope using stereotactic coordinates with a mouse matrix (Plastic One Inc.). Tissues were promptly collected and homogenized in ice‐cold RIPA buffer (9806S, Cell Signaling Technology) containing fresh protease inhibitors (4693116001, Roche) and phosphatase inhibitors (78420, Thermo Fisher). After homogenization, the samples were centrifuged at 12 000 g for 20 min at 4°C. The resulting supernatants were assayed for protein content, with protein concentration determined using the BCA method (23227, Thermo Fisher), and then diluted to equal protein concentration of 1.5 µg/µL. A total of 30 µg protein per sample was used to load onto 12% SDS polyacrylamide gel and then transferred onto PVDF membrane. The membrane was blocked with 5% BSA in 0.02% Teween‐20 tris‐buffered saline (TBST) for 1 h at room temperature and probed with primary antibodies (MBP, ab7349, Abcam; Hif‐3α, sc‐390933, santa cruz; Gapdh, 10494–1‐AP, Proteintech) at 4°C overnight and then incubated with HRP‐conjugated secondary antibodies [Goat anti‐rat IgG (1:1000; SA00001–15, Proteintech); Goat anti‐rabbit IgG (1:5000; A00098, GeneScript)] for 1 h at room temperature. The membrane was rinsed in TBST and scanned by an Imager (Bio‐Rad) after incubation with chemiluminescent detection reagent (36208ES60, Yeasen Biotech). The immunoblots were analyzed with ImageJ to measure the optical density of the bands of target proteins whose relative expression was calculated by normalizing the intensity of them to that of Gapdh.

### 5.7 Viral Constructs and Microinjection

Briefly, mice were anesthetized with isoflurane (3.5% induction, 1.5%–2% maintenance, RWD, China) and immobilized using a stereotactic device. A 0.5 mm burr hole was drilled into the skull. Vector particles were injected into the mPFC (anteroposterior, + 1.7 mm from bregma; mediolateral, ± 0.4 mm; dorsoventral, – 2.5 mm) using a glass electrode at a rate of 0.1 µL/ min in a total of 100 nL per side. When the injection was complete, the cannula was left to rest for 10 min to prevent efflux of the viral vector solution. The mice were allowed to recover for 3 weeks after surgery before further analysis. The interfering RNA sequences for knocking down NEPAS were antisense (5′‐TCACGCGCTGCAGCCCCAACGCCAT‐3′), sense (5′‐ATGGCGTTGGGGCTGCAGCGCGTGA‐3′), or scrambled oligonucleotides. For knocking down NEPAS in neurons and chemogenetic activation of mPFC neurons, the following AAV vectors were used: rAAV‐hSyn‐EGFP‐5'miR‐30a‐shRNA(Hif3α)‐miR30a‐WPRE, AAV2/9 (titer: 6 × 10^12^ vg/mL), rAAV‐hSyn‐EGFP‐5'miR‐30a‐shRNA(scramble)‐miR30a‐WPRE, AAV2/9 (titer: 5 × 10^12^ vg/mL), rAAV‐hSyn‐hM3D(Gq)‐EGFP‐WPRE‐hGH polyA, AAV2/9 (titer: 5 × 10^12^ vg/mL), rAAV‐hSyn‐mCherry‐WPRE‐hGH polyA, AAV2/9 (titer: 5 × 10^12^ vg/mL). For PTX3 overexpression in neurons, the following AAV vectors pAAV‐hSyn‐PTX3‐3×FLAG‐linker‐mCherry, AAV2/9 (titer: 5 × 10^12^ vg/mL), pAAV‐hSyn‐MCS‐mCherry, AAV2/9 (titer: 2.5 × 10^12^ vg/mL) were used. For in vivo fiber photometry recording, rAAV‐hSyn‐Gcamp6s‐WPRE‐hGH pA, AAV2/9 (titer: 5 × 10^12^ TU/mL) was injected into PVA (anteroposterior, + 1.7 mm from bregma; mediolateral, ± 0.4 mm; dorsoventral, ‐2.5 mm), and an optical fiber was implanted simultaneously. All rAAV and lentiviral vectors were constructed by BrainVTA, Wuhan.

### 5.8 In Vivo Fiber Photometry Recording

After a 3‐week recovery from surgery, fluorescence signals in PVA were recorded using a Fiber Photometry system equipped with 470‐ and 405‐nm excitation laser (Thinker Tech). The laser power at the tip of the optical fiber was adjusted to 1.8 to 2.0 µW for 405 nm and 18–20 µW for 470 nm by optical power meter. 470 nm (Ca^2+^‐dependent) and 405 nm (isosbestic reference fluorescence) fluorescence signals were collected by the CMOS at 40 Hz for single channel. Each mouse was recorded for 1 h one day before nicotine treatment (Pretest) and one day after nicotine WD (WD 7). The raw signals were adjusted to a flat baseline after baseline and motion correction using a script provided by Thinker Tech; the baseline‐adjusted signals were transformed as ΔF/F by dividing by their mean raw signals. The mice with off‐target fiber tips were excluded from the analysis.

### 5.9 The Novel Object Recognition Memory Task (NORT)

The novel object recognition test (ORT) is a straightforward memory assessment based on a rodent's innate exploratory behavior, where rodents tend to show a preference for exploring novel objects over familiar ones. An illuminated open field measuring 43 cm × 43 cm (L × W) was utilized for the ORT experiment. Mice underwent a 30‐min familiarization session daily in an empty arena over three days. During the training session, two identical Object A items were positioned at one‐fourth diagonal points within the arena. Mice were allowed to freely explore these objects for 5 min in four trials, with a 30‐min interval between each trial. On the memory test day, which occurred 24 h after the training session, one of the Object A items was substituted with a novel Object B. Mice were then given 5 min for free exploration of these objects. A video camera placed above the open field was connected to a video recorder and the mice travel distance was recorded by the SANS‐AI system (Sansbio, Jiangsu, China). Exploration of objects was defined as instances where the mice directed their noses toward the object, engaged in sniffing, or contacted the object using their snout or forepaws. Activities such as running around the object, sitting on it, or climbing on it were not considered exploration and were not recorded. The exploration time and entries were subsequently analyzed by a trained observer blind to the treatment according to the video by a stopwatch.

### 5.10 Conditioned Media Culture of Microvascular Endothelial Cells

To investigate the effect of neuronal NEPAS expression on the functions of microvascular endothelial cells in vitro, we separately cultured bEnd.3 cells (CL‐0598, Procell System, Wuhan, China) with media from the neuronal cell line HT‐22. HT‐22 cells were treated with lentivirus LV‐hsyn‐NEPAS‐2A‐EGFP‐WPRE (titer: 5 × 10^8^ TU/mL) or its control LV‐hsyn‐EGFP‐WPRE (titer: 5 × 10^8^ TU/mL) for 72 h followed by FACS sorting to obtain a stably NEPAS‐expressing cell line. After reaching confluence, the NEPAS‐stably expressing HT‐22 cells were seeded for 48 h, after which the culture supernatant was collected and centrifuged at 1,000 rpm for 1 min to remove cell debris. For the tube formation assay, the HT‐22 culture media was then added to bEnd.3 cells plated in Matrigel‐coated 24‐well plates. After 6 h of treatment, images of tube formation were captured via microscopy and analyzed using ImageJ software. For immunoblot and immunofluorescence analysis, bEnd.3 cells were treated with HT‐22 media for 48 h, after which cells or cell lysates were collected to assess the expression levels of ZO‐1 and Occludin.

### 5.11 Tube Formation Assay by bEnd.3 Cells

The angiogenic capacity of bEnd.3 cells were evaluated in vitro using a Matrigel‐based tube formation assay. LDEV‐Free Cultrex Basement Membrane Extract (40183ES08, Yeasen Biotech) was thawed overnight at 4°C and then coated onto 24‐well plates at 20 µL per well. The plates were incubated at 37°C for 30 min to allow gelation. bEnd.3 cells were resuspended in DMEM medium containing 10% FBS or experimental treatment medium at a density of 1 × 10^5^ cells per mL. The resulting suspensions were gently seeded onto the polymerized Matrigel surface and maintained at 37°C in a 5% CO_2_ humidified incubator for 6–8 h. Subsequently, the cells were stained with calcein AM for 30 min and imaged using a fluorescence inverted microscope. For investigating the effect of PTX3 on bEnd.3 angiogenic capacity, 100 nM recombinant mouse PTX3 (HY‐P71246, MedChemExpress) was added to the bEnd.3 cell culture medium, and tube formation was imaged after 8 h of further incubation. For quantitative analysis, 3 to 5 random fields of view were captured from each well, and the number of branches, number of junctions, and relative tube length were measured using the Angiogenesis Analyzer plugin in ImageJ. The values for each well were calculated as the mean of all fields of view within that well.

### 5.12 Statistical Analysis

Data consists of mean ± standard error of mean (SEM) and analyzed using GraphPad Prism 10.2.3. *P* < 0.05 was considered statistically significant. Comparisons between groups were analyzed by two‐tailed unpaired t‐test, one‐way ANOVA, or two‐way ANOVA, followed by the Tukey's post hoc test. Statistical significance was represented as ^*^ *P* < 0.05; ^**^ *P* < 0.01; ^***^ *P* < 0.001 and ^****^ *P* < 0.0001.

## Author Contributions

B.H. designed and directed the project; B.H., Z.C., X.J., B.S., J.G., J.X., and X.G. performed the experiments and collected the data; J.G., X.J., and Z.W. performed transcriptomic analysis; B.H., F.G., and P.L. analyzed the data and prepared figures; B.H. wrote the paper; and B.H., X.J., and J.W. revised the paper.

## Conflicts of Interest

The authors declare no conflicts of interest.

## Supporting information




**Supporting File**: advs75210‐sup‐0001‐SuppMat.docx.

**Supporting File**: advs75210‐sup‐0002‐Blots.zip.

## Data Availability

The data that support the findings of this study are available from the corresponding author upon reasonable request.
