# Supplementary material for: An Activity‐Dependent NEPAS–PTX3 Axis Links Neurovascular and Myelin Deficits to Cognitive Impairment
Source: Adv Sci (Weinh). 2026 Apr 7:e21069. Online ahead of print. doi: 10.1002/advs.202521069 (PMC13334678; doi:10.1002/advs.202521069)

((Supporting Information can be included here using this template))

Supporting Information

Title ((no stars))

An activity-dependent NEPAS–PTX3 axis links neurovascular and myelin deficits to cognitive impairment

*Boya Hu^2,†^, Zifei Chen^2,†^, Bingmei Sun^2,†^, Jiale Gao^2,†^, Jiale Xu^2^, Xiaochun Guo^2^, Fenfei Gao^4^, ZhongsiWang^5^, Jie Wu^1,2,3^, Xiaoyu Ji^1,2,3,*^, Peipei Liu^6^**^,*^, Bing Huang^1,2,3,4,*^*


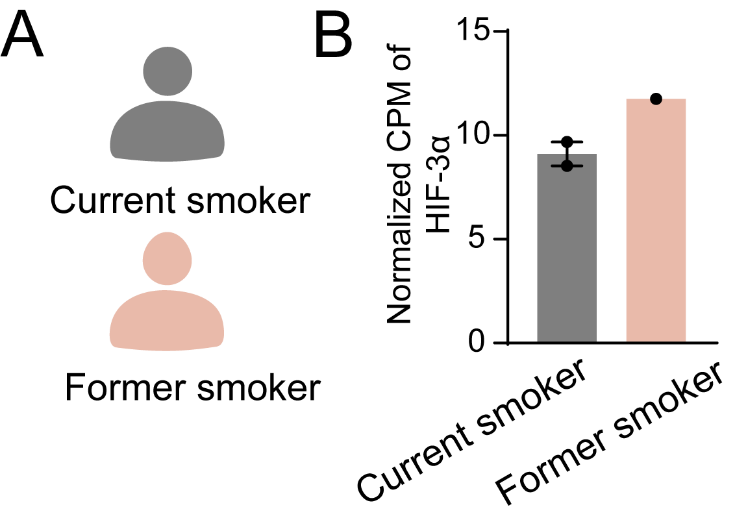


**Figure S1. Analysis of the transcription level of HIF-3α in postmortem prefrontal cortex of a human cohort.** (A) Transcriptomic data from two current smokers and one former smoker who had undergone smoking cessation were used in this analysis. (B) Counts per million showed a higher trend in HIF-3α mRNA levels in the former smoker compared to the current smokers.


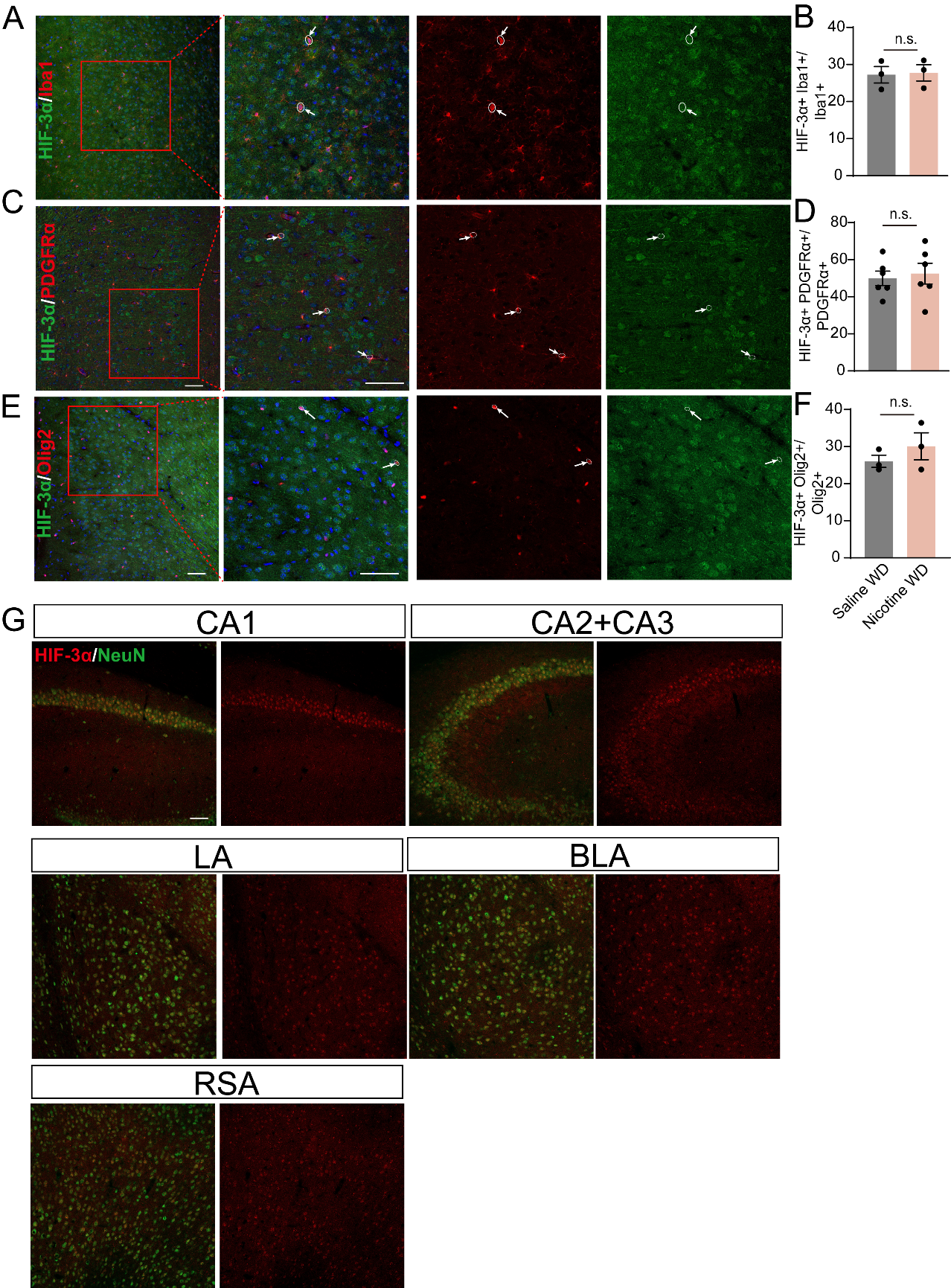
**Figure S2. Nicotine WD had no effect on the expression of HIF-3α in glia cells.** (A-B) Immunofluorescence images showing co-staining of Iba1 and HIF-3α revealed no significant change in HIF-3α expression in Iba1-positive cells following nicotine WD (n = 3 for each group; ^*^*P* = 0.8774, two-tailed Student’s t test). (C-D) Immunofluorescence images showing co-staining of PDGFRα and HIF-3α revealed no significant change in HIF-3α expression in PDGFRα-positive cells following nicotine WD (n = 6 for each group; ^*^*P* = 0.7170, two-tailed Student’s t test). (E-F) Immunofluorescence images showing co-staining of Olig2 and HIF-3α revealed no significant change in HIF-3α expression in Olig2-positive cells following nicotine WD (n = 3 for each group; ^*^*P* = 0.3693, two-tailed Student’s t test). (G) HIF-3α expressed in neurons of various regions, including hippocampus (CA1, CA1, CA3), amygdala (LA, BLA) and agranular cortex (RSA) etc. BLA, basolateral amygdala; LA, lateral amygdala; RSA, retrosplenial agranular cortex.


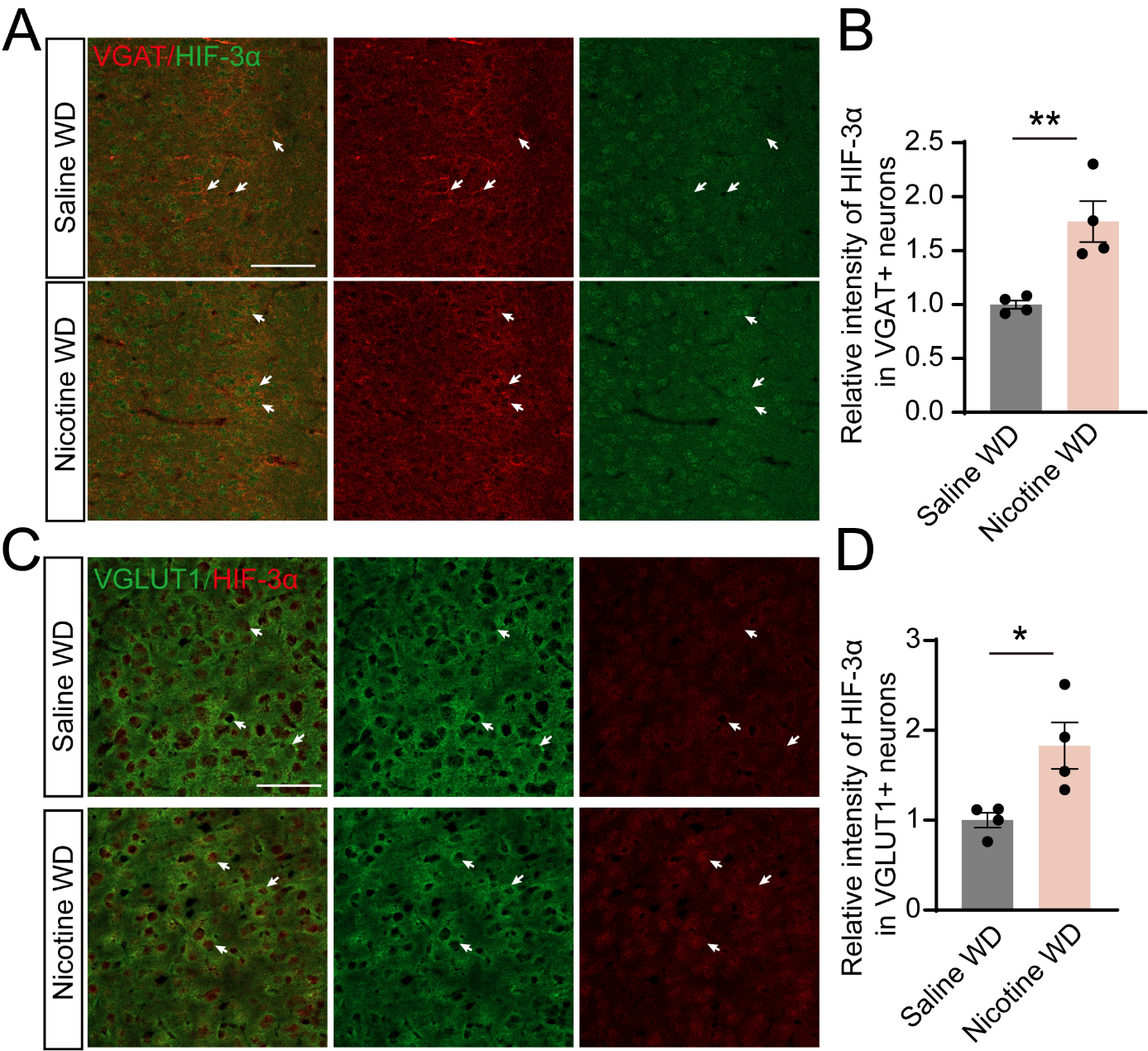


**Figure S3. Expression of HIF-3α was upregulated in mPFC neurons innervated by glutermatergic and GABAergic afferent after nicotine WD.** (A-B) Immunofluorescence images of co-staining of HIF-3α and VGAT revealed a significant increase in HIF-3α expression in VGAT innervated neurons following nicotine WD (n = 4 for each group; ^**^*P* = 0.0074, two-tailed Student’s t test). (C-D) Immunofluorescence images of co-staining of HIF-3α and VGLUT1 revealed a significant increase in HIF-3α expression in VGLUT1 innervated neurons following nicotine WD (n = 4 for each group; ^*^*P* = 0.0223, two-tailed Student’s t test).


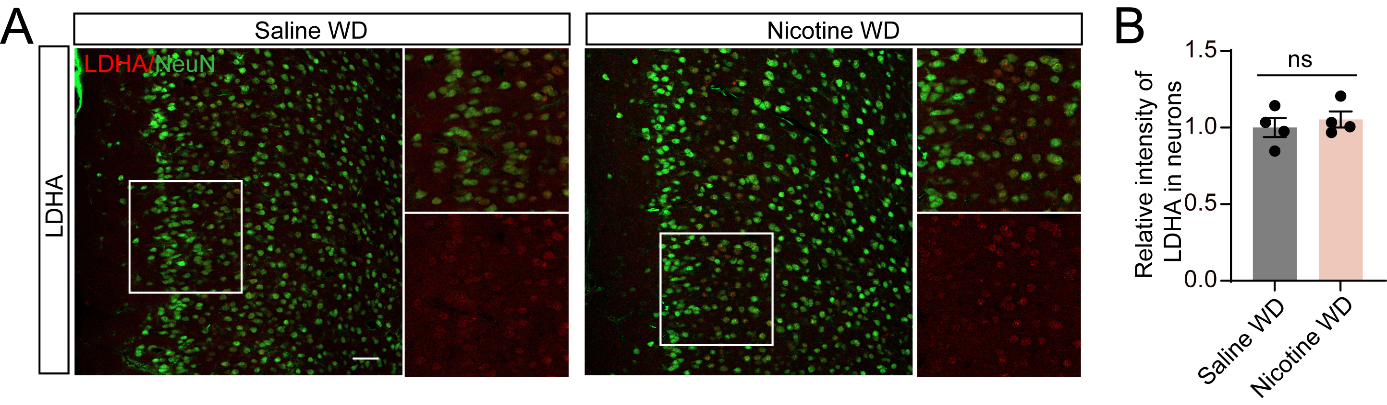


**Figure S4. Nicotine WD had no effect on the expression of LDHA in neurons.** (A) Immunofluorescence staining of LDHA in neurons. (B) Statistic results showed no difference in the expression of LDHA between the saline WD and nicotine WD groups (n = 4 for each group; ^*^*P* = 0.5367, two-tailed Student’s t test). Scale bars, 50 μm. Data are presented as mean ± SEM. LDHA, Lactate dehydrogenase A.


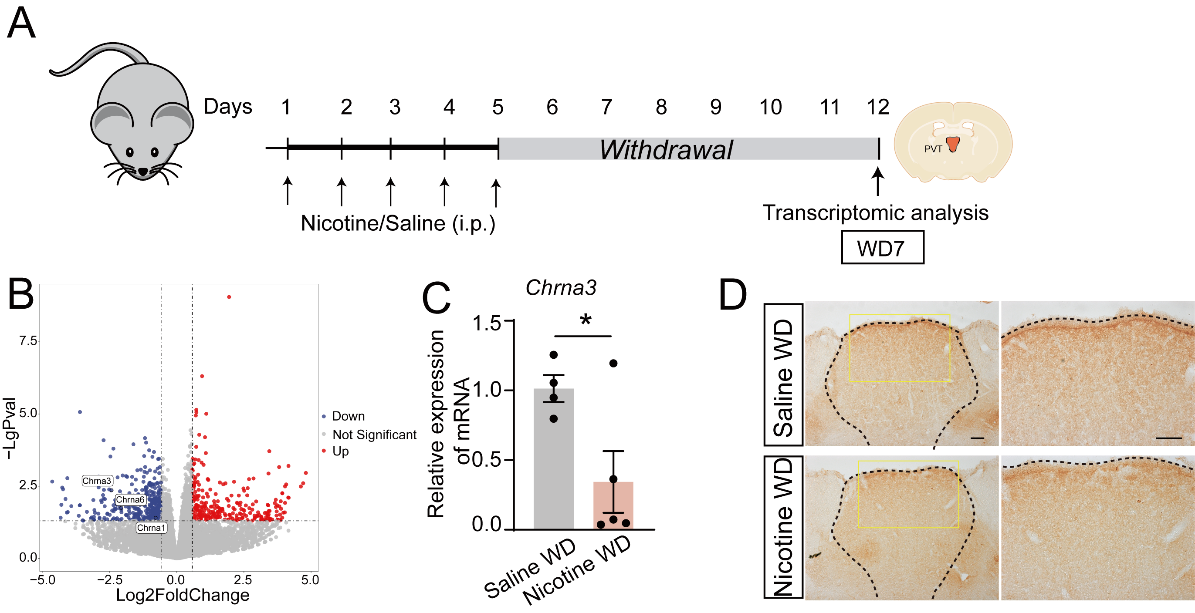
**Figure S5. Expression of chrna3 in PVA was downregulated after nicotine WD.** (A) Schematic diagram displaying the time course and brain region of transcriptomic analysis. (B) Volcano plot showed DEGs between nicotine WD and saline WD, with *chrna1, chrna3, and chrna6* were downregulated. (C) RT-qPCR showed a decrease in mRNA of *chrna3* (n = 4,5 for Saline WD and Nicotine WD, respectively; ^*^*P* < 0.05, two-tailed Student’s t test). (D) Immunohistochemistry image of CHRNA3 expression in PVA.


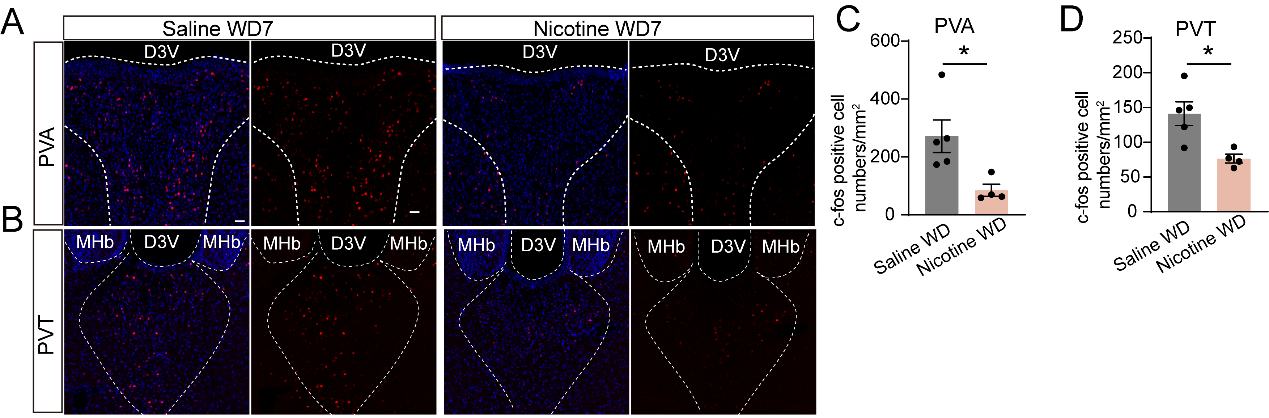


**Figure S6. Nicotine WD caused suppressed neuronal activities in the PVA.** (A) Representative images (A-B) and statistics analysis (C-D) of c-fos expression in both aPVT and pPVT, with a decreased c-fos expression in both regions (n = 5, 4 for Saline WD and Nicotine WD, respectively; ^*^*P* < 0.05, two-tailed Student’s t test). Scale bars, 50 μm. Data are presented as mean ± SEM.


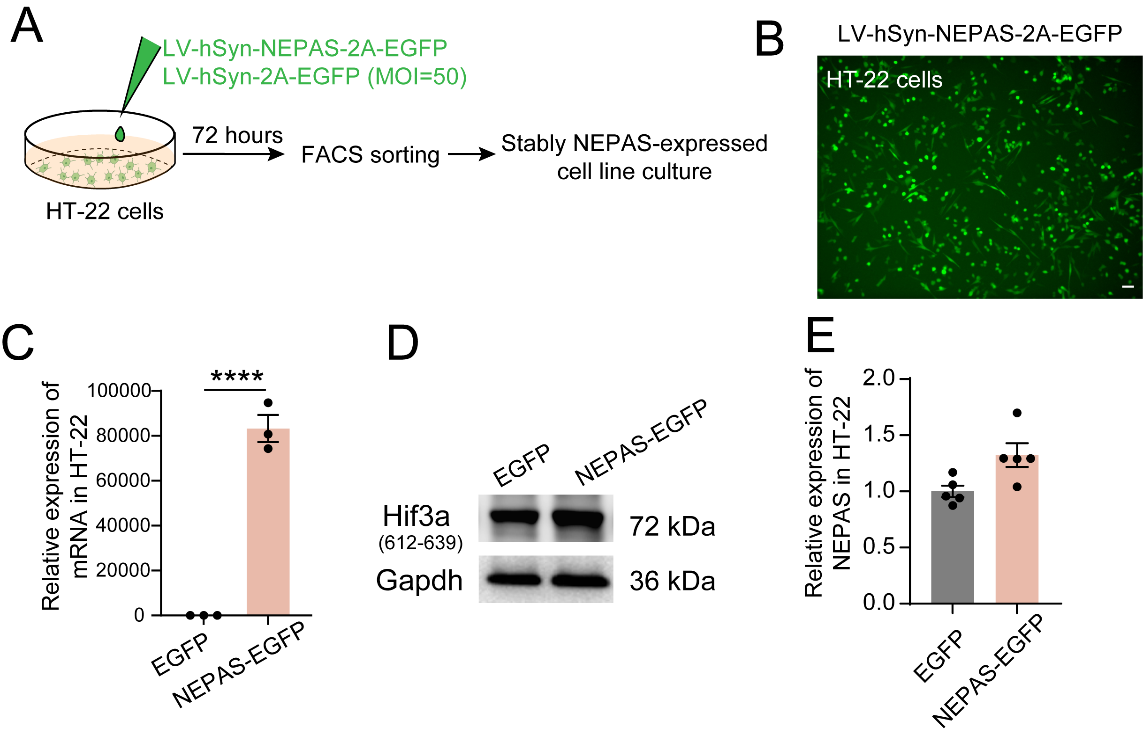


**Figure S7. Generation of NEPAS stably overexpressed neuronal cell line.** (A) HT-22 cells were transfected with lentivirus carried NEPAS overexpression vector (LV-hSyn-NEPAS-2A-EGFP) or its control LV-hSyn-2A-EGFP with MOI=50. GFP signals began to appear in the cells 72 hours after transfection and NEPAS stably overexpressed cells were obtained by FACS sorting through GFP signals. Stable cell lines were cultured for further assays. (B) Representative image of NEPAS stably overexpressed HT-22 cell line in culture. (C) The NEPAS mRNA level in HT-22 cells was significantly increased in NEPAS-EGFP group (n = 3 for each group, ^****^*P* < 0.0001, two-tailed Student’s t test). (D-E) Immunoblotting showed that the protein of NEPAS in HT-22 cells was increased after overexpression (n=5 for each group, ^*^*P* < 0.05, two-tailed Student’s t test). Data are presented as mean ± SEM.


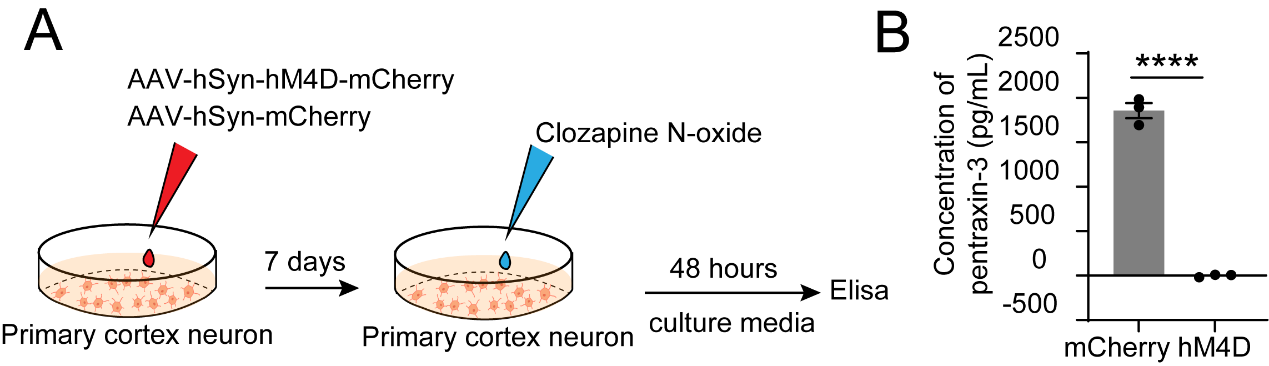


**Figure S8. Release of PTX3 was dependent on neuronal activity.** (A) Primary cortical neurons were cultured in vitro for 5 days, then transfected with hSyn-hM4D-mCherry or the control vector hSyn-mCherry for 7 days, followed by 2 days of CNO treatment to induce neuronal inactivation. Culture medium was subsequently collected to assess PTX3 secretion. (B) PTX3 secretion from inactivated neurons was dramatically reduced compared to the control group (n = 3 for each group, ^****^*P* < 0.0001, two-tailed Student’s t test).


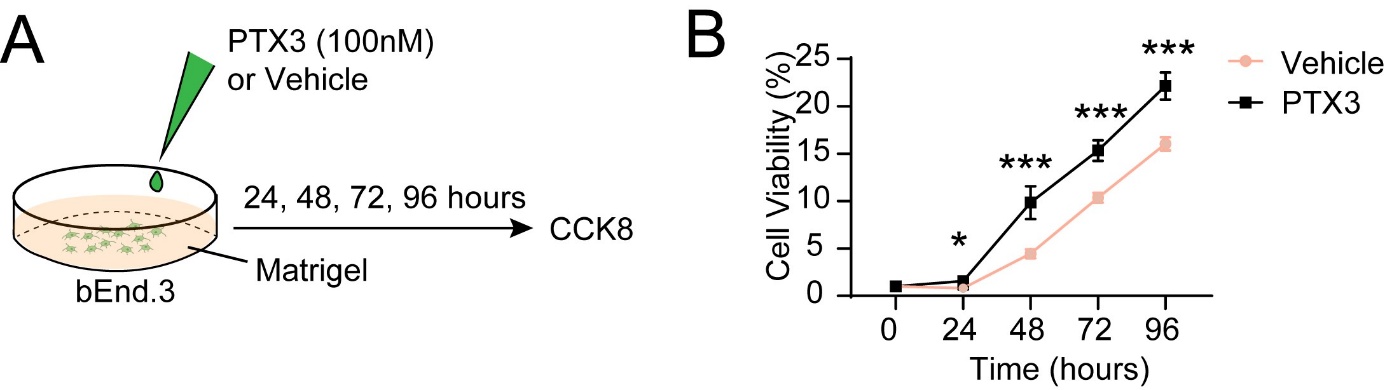


**Figure S9. PTX3 enhanced proliferation rate of bEnd.3.** (A) Schematic diagram displaying PTX3 treatment and timepoints for detecting proliferation rate by CCK-8. (B) PTX3 significantly promoted the proliferation rate of bEnd.3 after treating for 24, 48, 72, and 96 h (n = 4 for each group, ^*^*P* < 0.05, ^***^*P* < 0.001, two-tailed Student’s t test). Data are presented as mean ± SEM.


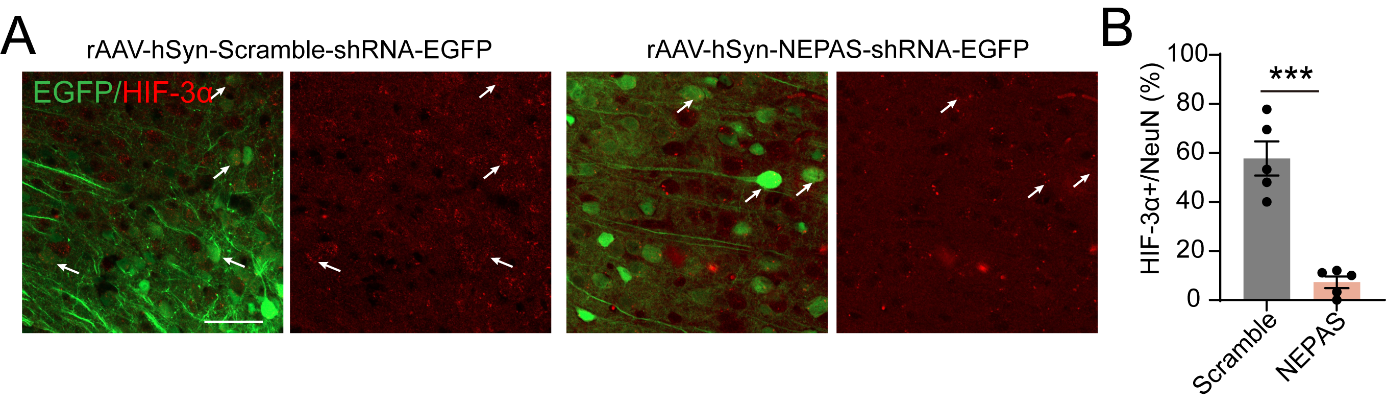


**Figure S10. Expression of neuronal NEPAS was decreased following shRNA injection.** (A) Immunofluorescence images showed expression of HIF-3α in neurons. (B) Quantitative data showing reduced NEPAS expression in neurons after NEPAS shRNA injection compared to scramble shRNA control (n = 5 for each group, ^***^*P* < 0.001, two-tailed Student’s t test).


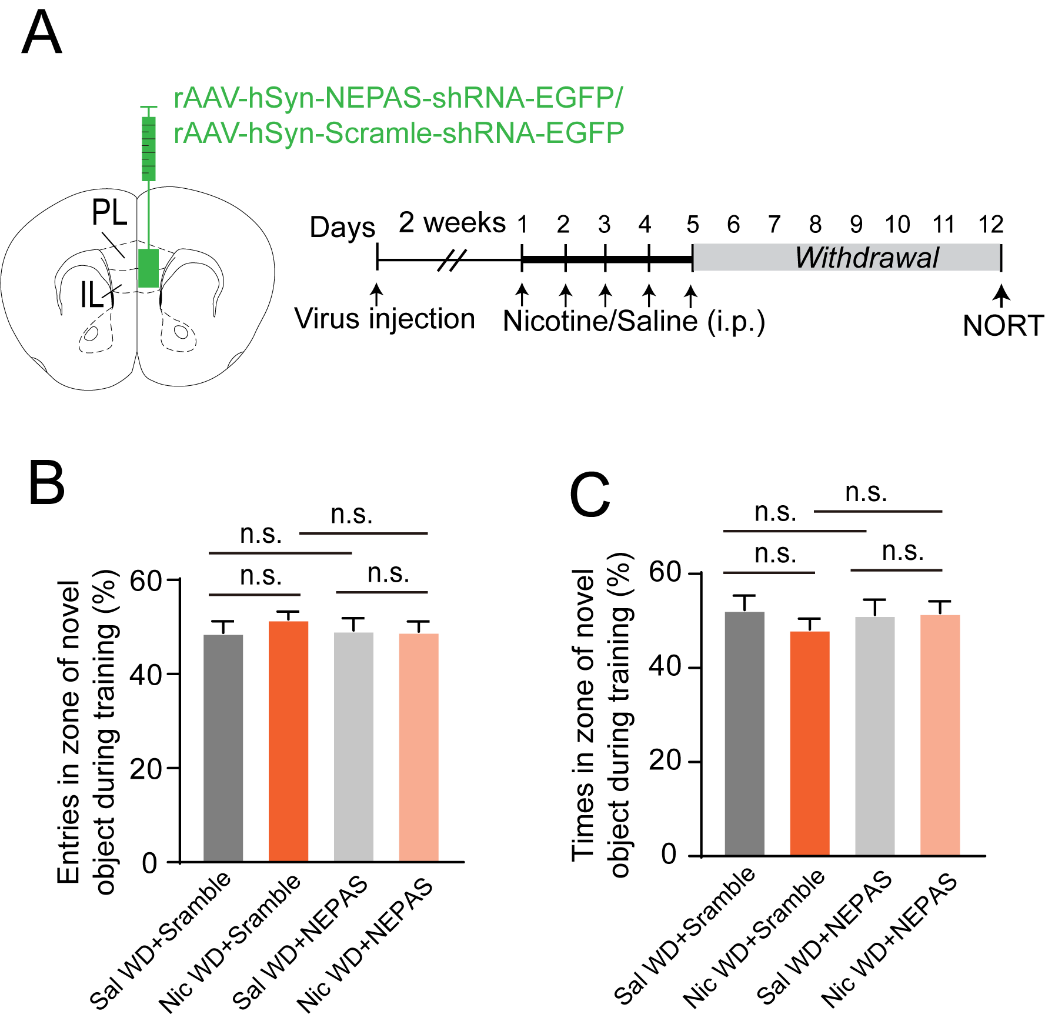


**Figure S11. Knocking down of neuronal NEPAS had no effect on learning ability in NORT task.** (A) Schematic diagram displaying AAV injection of hSyn-NEPAS-shRNA or control hSyn-scramble-shRNA into mPFC. After a 2-week recovery, mice were conducted to nicotine WD procedure, followed by NORT task. (B) Statistics results showed no significant differences in entries to zone B among each group (n=12, 12, 11, 11 for Sal WD + Scramble, Nic WD + Scramble, Sal WD + NEPAS, and Nic WD + NEPAS, respectively; F (3,42) = 0.358, *P* = 0.7837, two-way ANOVA with Tukey’s post hoc test). (C) Statistics results showed no significant differences in exploration times to zone B among each group (n=12, 12, 11, 11 for Sal WD + Scramble, Nic WD + Scramble, Sal WD + NEPAS, and Nic WD + NEPAS, respectively; F (3,42) = 0.457, *P* = 0.7137, two-way ANOVA with Tukey’s post hoc test). Data are presented as mean ± SEM.


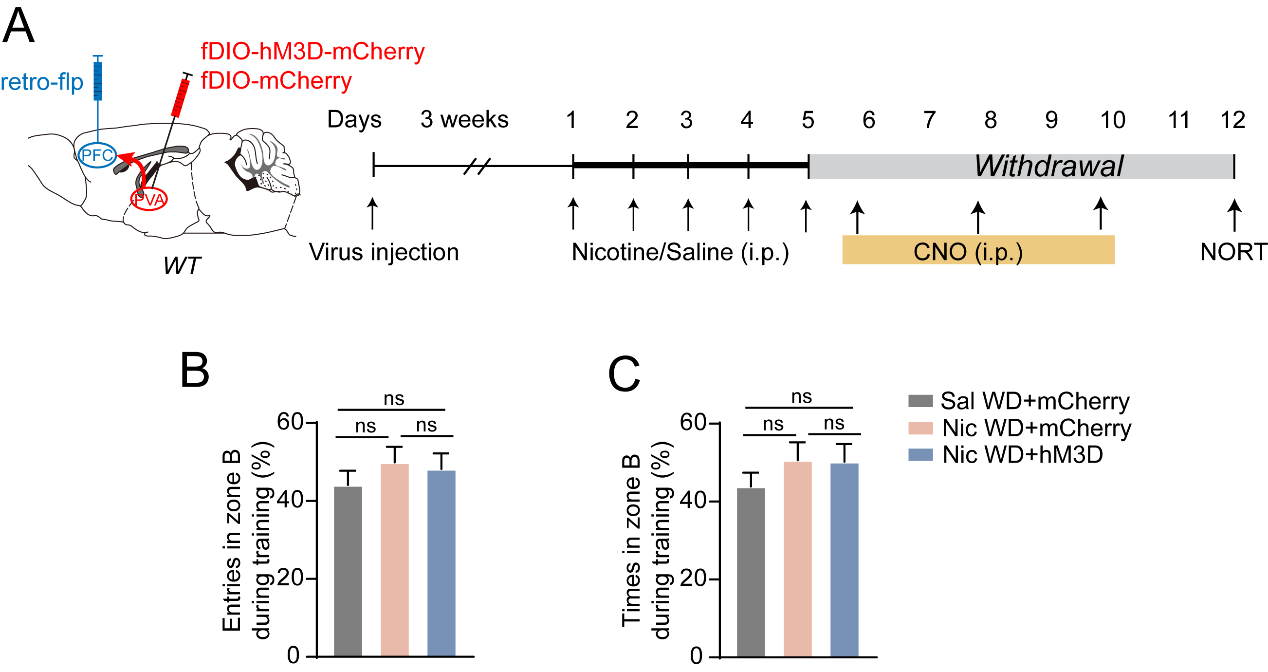


**Figure S12. Chemogenetics activation of mPFC neurons innervated by PVA neurons had no effect on learning ability in NORT task.** (A) Schematic diagram displaying AAV injection strategies and chemogenetics manipulation procedure in WT mice. AAV-hSyn-flp (retro) was injected into mPFC, and AAV-hSyn-fDIO-hM3D-mCherry or AAV-hSyn-fDIO-mCherry were injected to PVA. After a 3-week recovery, mice were conducted to nicotine WD procedure, followed by NORT task. (B) Activation of mPFC neurons innervated by PVA neurons had no effect on the entries in zone B (n=11 for each group, F (2,30) =0.596, *P* = 0.5576; Sal WD + mCherry vs. Nic WD + mCherry, *P* = 0.5440; Nic WD + mCherry vs. Nic WD + hM3D, *P* =0.9465, one-way ANOVA with Tukey’s post hoc test). (C) Activation of these mPFC neurons had no effect on the exploration times to zone B (n=11 for each group, F (2,30) =0.795, *P* = 0.4607; Sal WD + mCherry vs. Nic WD + mCherry, *P* = 0.5087; Nic WD + mCherry vs. Nic WD + hM3D, *P* = 0.9980, one-way ANOVA with Tukey’s post hoc test). Data are presented as mean ± SEM.

**Summary statistics for each experimental group.**

| **Figures** | **Group** | | **mean±SEM** | **n** | ***P*** |
| --- | --- | --- | --- | --- | --- |
| Fig. 1C | HIF-3α | Saline WD  Nicotine WD | 0.000 ± 0.000  0.031 ± 0.018 | 4  4 | 0.134 |
|  | NEPAS  /IPAS | Saline WD  Nicotine WD | 1.334 ± 0.058  2.958 ± 0.515 | 4  4 | 0.020 |
| Fig. 1D |  | Saline WD  Nicotine WD | 1.025 ± 0.126  2.110 ± 0.365 | 4  4 | 0.0306 |
| Fig. 1E |  | Saline WD  Nicotine WD | 1.102 ± 0.221  2.489 ± 0.192 | 5  5 | 0.0015 |
| Fig. 1F |  | Saline WD  Nicotine WD | 1.106 ± 0.260  1.264 ± 0.322 | 3  4 | 0.6614 |
| Fig. 1H |  | Saline WD  Nicotine WD | 1.000 ± 0.170  1.769 ± 0.212 | 6  6 | 0.0179 |
| Fig. 1J |  | Saline WD  Nicotine WD | 65.50 ± 3.159  78.82 ± 1.810 | 4  4 | 0.0064 |
| Fig. 1M |  | Saline WD  Nicotine WD | 6.384 ± 0.685  3.612 ± 0.366 | 6  7 | 0.0034 |
| Fig. 2B |  | Saline WD  Nicotine WD | 17.16 ± 1.375  9.405 ± 0.699 | 5  4 | 0.0070 |
| Fig. 2C |  | Saline WD  Nicotine WD | 13.29 ± 1.033  7.288 ± 1.416 | 5  4 | 0.0098 |
| Fig. 2E |  | Saline WD  Nicotine WD | 1.014 ± 0.189  0.501 ± 0.016 | 4  4 | 0.0394 |
| Fig. 2I |  | Saline WD  Nicotine WD | 1.000 ± 0.189  0.356 ± 0.098 | 5  6 | 0.011 |
| Fig. 2N |  | Sal WD + mCherry  Nic WD + mCherry  Nic WD + hM3D | 16.82 ± 1.700  7.580 ± 0.901  20.93 ± 3.202 | 4  4  4 | 0.0058 |
| Fig. 2P |  | Sal WD + mCherry  Nic WD + mCherry  Nic WD + hM3D | 1.000 ± 0.047  1.429 ± 0.096  0.822 ± 0.102 | 4  5  5 | 0.0014 |
| Fig. 3B |  | Saline WD  Nicotine WD | 1.000 ± 0.068  0.481 ± 0.166 | 4  4 | 0.0276 |
| Fig. 3C |  | Saline WD  Nicotine WD | 16.25 ± 1.652  6.500 ± 2.179 | 4  4 | 0.0119 |
| Fig. 3F |  | EGFP  NEPAS-EGFP | 60.50 ± 8.319  14.77 ± 3.638 | 12  13 | < 0.0001 |
| Fig. 3G |  | EGFP  NEPAS-EGFP | 50.83 ± 4.039  23.85 ± 3.956 | 12  13 | < 0.0001 |
| Fig. 3H |  | EGFP  NEPAS-EGFP | 1.000 ± 0.145  0.2682 ± 0.052 | 12  13 | < 0.0001 |
| Fig. 3J |  | EGFP  NEPAS-EGFP | 1.000 ± 0.0814  0.813 ± 0.013 | 8  8 | 0.0397 |
| Fig. 3L |  | EGFP  NEPAS-EGFP | 1.000 ± 0.057  0.771 ± 0.063 | 8  8 | 0.0176 |
| Fig. 3N |  | EGFP  NEPAS-EGFP | 5165 ± 42.79  4482 ± 73.45 | 4  4 | 0.0002 |
| Fig. 3P |  | Vehicle  PTX3 | 40.40 ± 4.287  148.3 ± 3.995 | 10  10 | < 0.0001 |
| Fig. 3Q |  | Vehicle  PTX3 | 48.00 ± 4.161  87.90 ± 4.119 | 10  10 | < 0.0001 |
| Fig. 3R |  | Vehicle  PTX3 | 1.000 ± 0.105  3.667 ± 0.069 | 10  10 | < 0.0001 |
| Fig. 3T |  | mCherry  PTX3 | \| 0.9686 ± 0.042   \| \| 1.379 ± 0.052 \| \| --- \| \| \| --- \| --- \| \| \| --- \| --- \| --- \| | 3  3 | 0.0036 |
| Fig. 4C |  | Scramble  NEPAS | 1.000±0.099  2.620±0.190 | 4  4 | 0.0003 |
| Fig. 4E |  | Sal WD + Scramble  Nic WD + Scramble  Sal WD + NEPAS  Nic WD + NEPAS | 1.000 ± 0.082  0.439 ± 0.052  0.804 ± 0.133  0.755 ± 0.055 | 5  4  3  3 | 0.0124 |
| Fig. 4G |  | Sal WD + Scramble  Nic WD + Scramble  Sal WD + NEPAS  Nic WD + NEPAS | 1.000 ± 0.059  0.777 ± 0.049  0.848 ± 0.047  0.9825 ± 0.008 | 5  4  3  3 | 0.0063 |
| Fig. 4J |  | Sal WD + Scramble  Nic WD + Scramble  Sal WD + NEPAS  Nic WD + NEPAS | 66.30 ± 3.683  51.50 ± 2.288  61.98 ± 2.837  64.54 ± 3.187 | 12  12  11  11 | 0.0053 |
| Fig. 4K |  | Sal WD + Scramble  Nic WD + Scramble  Sal WD + NEPAS  Nic WD + NEPAS | 82.11 ± 2.293  68.82 ± 2.620  77.00 ± 1.975  78.55 ± 2.680 | 12  12  11  11 | 0.0024 |
| Fig. 5C |  | Sal WD + mCherry  Nic WD + mCherry  Nic WD + hM3D | 1.000 ± 0.077  0.592 ± 0.143  3.260 ± 0.615 | 6  6  4 | < 0.0001 |
| Fig. 5E |  | Sal WD + mCherry  Nic WD + mCherry  Nic WD + hM3D | 1.000 ± 0.063  0.730 ± 0.045  0.823 ± 0.075 | 4  5  5 | 0.0372 |
| Fig. 5G |  | Sal WD + mCherry  Nic WD + mCherry  Nic WD + hM3D | 6.154 ± 0.557  2.291 ± 0.325  5.360 ± 0.791 | 4  4  4 | 0.0029 |
| Fig. 5J |  | Sal WD + mCherry  Nic WD + mCherry  Nic WD + hM3D | 61.86 ± 1.878  53.52 ± 2.269  66.52 ± 2.343 | 11  11  11 | 0.0008 |
| Fig. 5K |  | Sal WD + mCherry  Nic WD + mCherry  Nic WD + hM3D | 80.67 ± 2.067  70.99 ± 3.091  85.61 ± 1.672 | 11  11  11 | 0.0005 |

**Uncropped and unprocessed original gel and blot images used in the manuscript.**


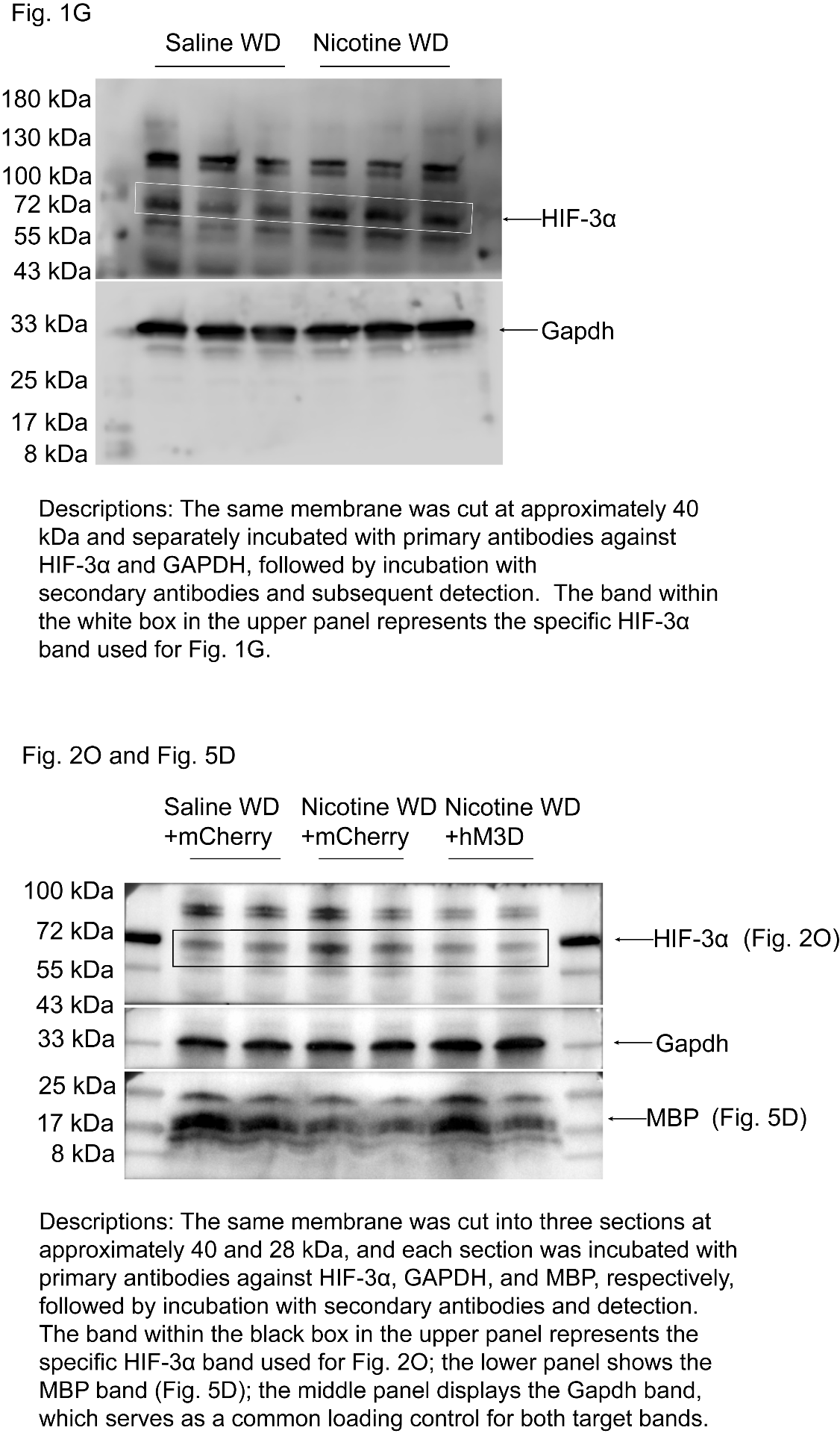

Supplement: Supplementary file 1 — Supporting File: advs75210‐sup‐0001‐SuppMat.docx. [file ADVS-9999-e21069-s002.docx]
