# Supplementary figures and images for: An Activity‐Dependent NEPAS–PTX3 Axis Links Neurovascular and Myelin Deficits to Cognitive Impairment
Source: Adv Sci (Weinh). 2026 Apr 7:e21069. Online ahead of print. doi: 10.1002/advs.202521069 (PMC13334678; doi:10.1002/advs.202521069)

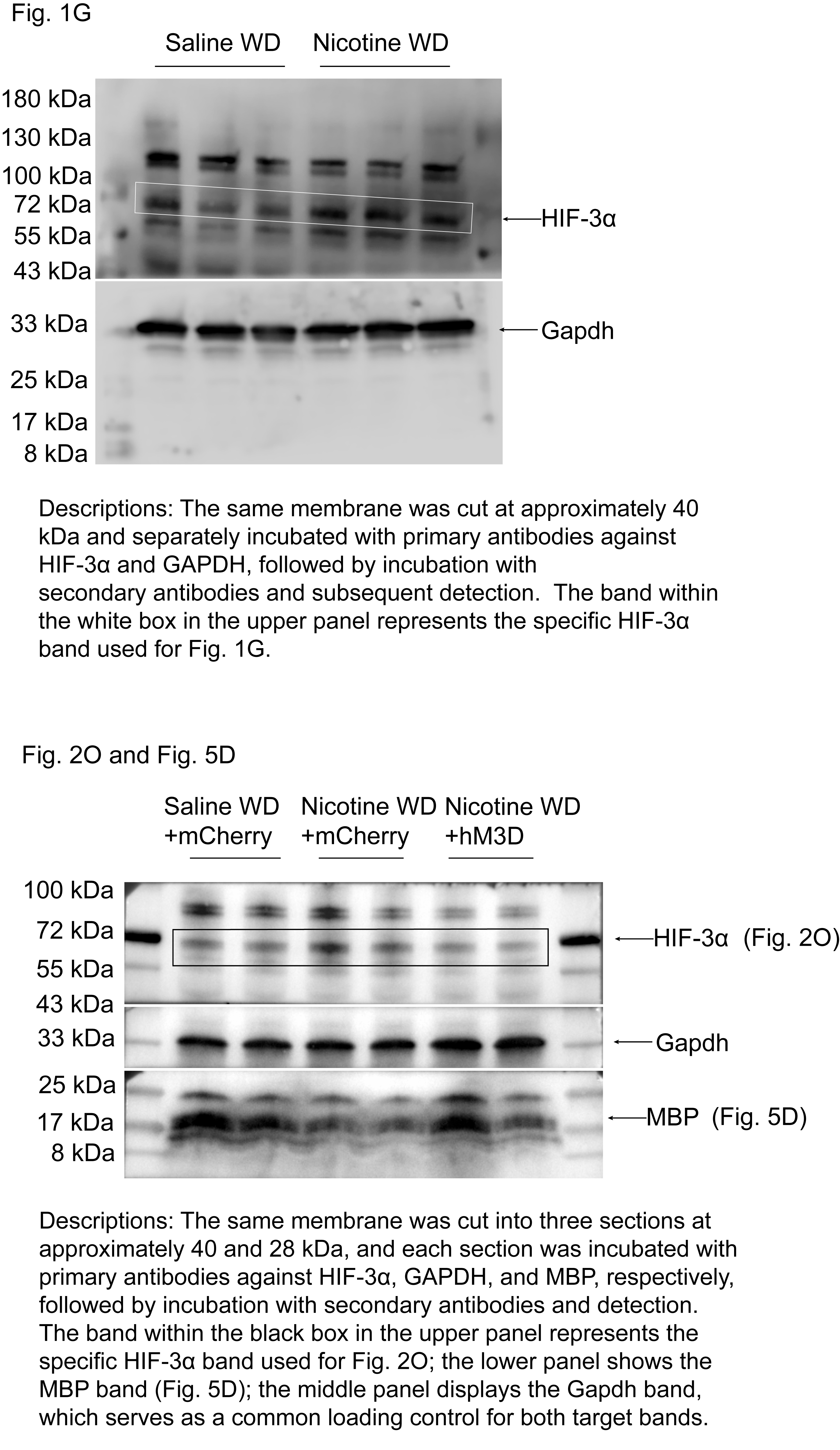

Supplement: Supplementary file 2 — Supporting File: advs75210‐sup‐0002‐Blots.zip. [file ADVS-9999-e21069-s001.zip › advs75210-sup-0002-Blots/Full-length blots.tif]
